# Ionic liquid-containing cathodes empowering ceramic solid electrolytes

**DOI:** 10.1016/j.isci.2022.103896

**Published:** 2022-02-11

**Authors:** Eric Jianfeng Cheng, Mao Shoji, Takeshi Abe, Kiyoshi Kanamura

**Affiliations:** 1 Graduate School of Engineering, Kyoto University, Kyoto 615-8510, Japan; 2 Graduate School of Urban Environmental Sciences, Tokyo Metropolitan University, Tokyo 192-0397, Japan

**Keywords:** Energy storage, Materials science, Ceramics

## Abstract

Although ceramic solid electrolytes, such as Li_7_La_3_Zr_2_O_12_ (LLZO), are promising candidates to replace conventional liquid electrolytes for developing safe and high-energy-density solid-state Li-metal batteries, the large interfacial resistance between cathodes and ceramic solid electrolytes severely limits their practical application. Here we developed an ionic liquid (IL)-containing while nonfluidic quasi-solid-state LiCoO_2_ (LCO) composite cathode, which can maintain good contact with an Al-doped LLZO (Al-LLZO) ceramic electrolyte. Accordingly the interfacial resistance between LCO and Al-LLZO was significantly decreased. Quasi-solid-state LCO/Al-LLZO/Li cells demonstrated relatively high capacity retention of about 80% after 100 cycles at 60°C. The capacity decay was mainly because of the instability of the IL. Nevertheless, the IL-containing LCO cathode enabled the use of Al-LLZO as a solid electrolyte in a simple and practical way. Identifying a suitable IL is critical for the development of quasi-solid-state Li-metal batteries with a ceramic solid electrolyte.

## Highlights

• An ionic liquid (IL)-containing quasi-solid-state LiCoO_2_ (LCO) cathode was developed
• Quasi-solid-state LCO/Al-doped Li_7_La_3_Zr_2_O_12_/Li cell showed high capacity retention
• Optimal IL content in the quasi-solid-state LCO cathode was about 11 wt%
• Battery capacity degradation was mainly due to the instability of the IL

## Graphical abstract

**Figure:**
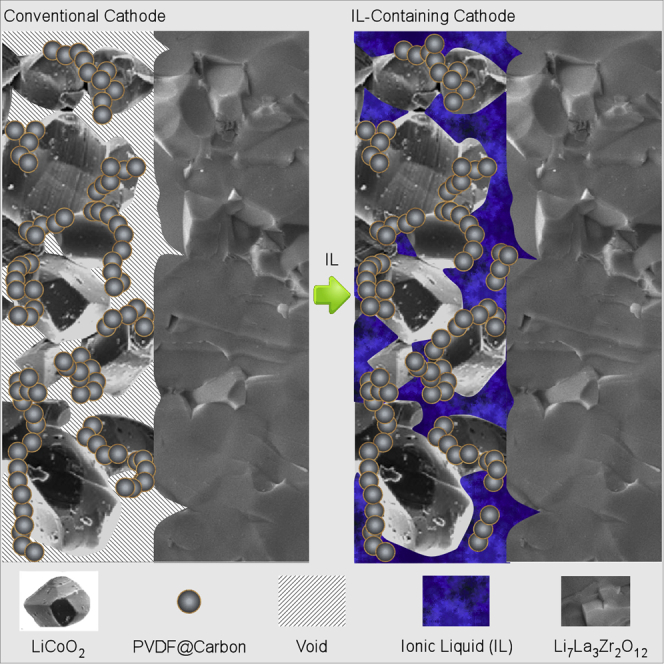

## Introduction

Owing to the unmatched energy density, rechargeable Li-ion batteries have dominated the portable electronics market for three decades since their first commercialization by Sony in 1991 (Yoshino, 2012). However, the emerging electric vehicle (EV) industry is concerned with maximizing the driving range and improving the safety of the vehicles (Van Noorden, 2014; Cui, 2020) Advanced batteries are not only critical for EVs but are also essential in integrating renewable energy resources, such as wind and solar energies, into the electric power grid to supplement the growing energy demand worldwide. Because a Li metal anode has an ultrahigh theoretical specific capacity of 3860 mA h g^−1^, about 10 times higher than that of a graphite anode, and the most negative redox potential of −3.04 V versus standard hydrogen electrode (SHE), Li-metal batteries have regained tremendous interest recently as promising next-generation energy storage devices (Fang et al., 2019).

However, the practical use of Li metal anode is challenging because of uncontrollable Li dendrite growth and severe side reactions between Li metal and conventional liquid electrolytes. Li metal was first reported to be used as an anode material in the TiS_2_-Li rechargeable battery in 1976 by Stanley Whittingham (1976), but was abandoned thereafter because of severe Li dendrite growth, which not only caused rapid capacity decay but also posed an explosion hazard. Unlike liquid electrolytes, solid electrolytes have the potential to physically suppress the initiation and propagation of Li dendrite growth, because they have relatively high elastic and shear moduli (Monroe and Newman, 2005; Yu et al., 2016; Krauskopf et al., 2020). Though many solid electrolytes exhibit fast-ion conductivity (e.g., 10^−3^ S cm^−1^ at 25°C), few are stable against Li metal. The garnet-type solid electrolyte, LLZO, is widely regarded as a promising solid electrolyte because of its high ionic conductivity and high chemical stability against Li metal (Murugan et al., 2007; Zhao et al., 2019). Although sulfide solid electrolytes, such as Li_2_S-P_2_S_5_, usually have higher ionic conductivities than LLZO, they must be handled in air-free conditions because hydrolysis of sulfide electrolytes by water vapor in air generates smelly and toxic H_2_S gas (Tatsumisago et al., 2013). In comparison, LLZO is relatively stable in air, odorless, and nonflammable.

Nonetheless, it is difficult to form good interfacial contact between LLZO and electrode materials because high-temperature sintered LLZO is rigid and brittle (Vickers hardness: ～6.3 GPa) (Yu et al., 2016; Famprikis et al., 2019; Ni et al., 2012). This results in high interfacial resistance, preventing the practical use of LLZO as a promising solid electrolyte (Kim et al., 2021; Wang et al., 2020a). A tremendous research effort has been devoted to reducing the high interfacial resistance in solid-state Li metal batteries with an LLZO electrolyte. For example, ultralow interfacial resistance, as low as 1 Ω cm^2^, could be achieved by introducing a thin Au, Ag, ZnO, or Al_2_O_3_ interlayer into the LLZO/Li interface (Han et al., 2017; Wakasugi et al., 2017; Tsai et al., 2016; Feng et al., 2019; Wang et al., 2017). Good interfacial contact between LLZO and Li metal can also be achieved by high pressure and heat treatment (Inada et al., 2018; Zheng et al., 2019; Wang et al., 2019) because Li metal is soft and has a relatively low melting point of 180.5°C. In contrast, much less effort has been devoted to reducing the interfacial resistance between LLZO and the cathode materials. Although good interfacial bonding between LLZO and LCO could be achieved by co-sintering (Ohta et al., 2014), LCO is likely to decompose at temperatures greater than 900°C (Cheng et al., 2017) and Li loss and various side reactions are also likely to occur at high temperatures.

**Figure 1:**
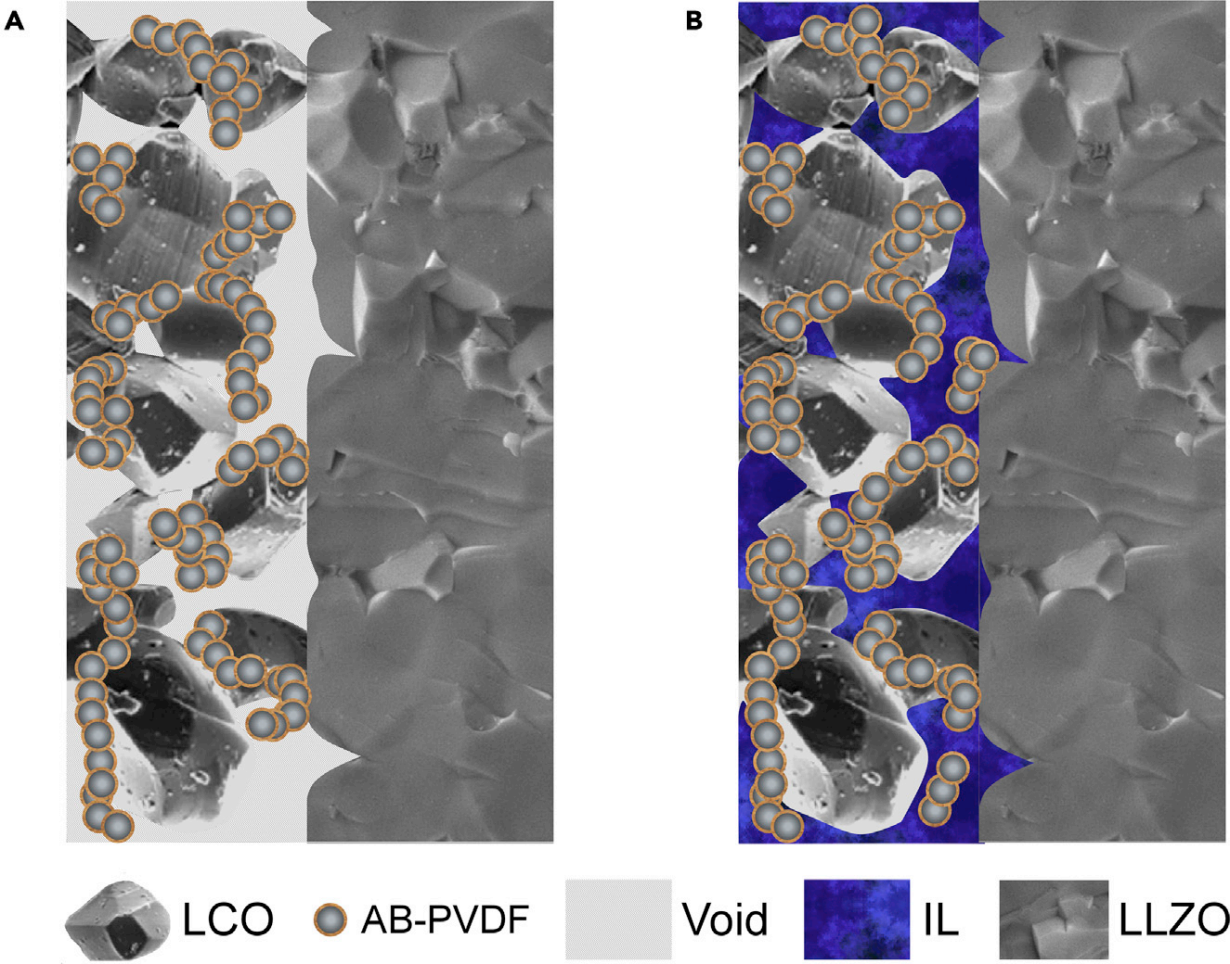
Illustration of an LCO cathode with and without an IL (A) without an IL, (B) with an IL. The LCO/LLZO interface is wetted by the IL.

A convenient and effective way to construct a conformal interface for fast Li-ion transport is to introduce a small amount of liquid electrolyte to wet the cathode/solid electrolyte interface (Cheng et al., 2020; Zhao et al., 2020). Because room temperature ILs are highly conductive, thermally stable, almost nonvolatile, and nonflammable, they are ideal candidates for wetting the cathode/solid electrolyte interface (Watanabe et al., 2017). For example, LCO/Li cells with a quasi-solid-state IL-containing LLZO electrolyte demonstrated a high initial discharge capacity of 130 mA h g^−1^ and high capacity retention of 99% after 150 cycles (Kim et al., 2016). However, the quasi-solid-state IL-containing LLZO electrolyte was fragile and difficult to handle. Similarly, an IL can be introduced into a cathode to form a quasi-solid-state composite cathode. A conventional cathode slurry usually consists of an active material (e.g., LCO), a polymer binder (e.g., polyvinylidene difluoride (PVDF)), a conductive agent (e.g., acetylene black (AB)), and a solvent (e.g., N-methyl-2-pyrrolidone (NMP)). After the evaporation of NMP, the physical contact between an LCO cathode and an LLZO electrolyte would mainly be point-to-point contact, as illustrated in Figure 1A. However, the cathode/solid electrolyte interface can be wetted by a small amount of IL, which can be introduced into the cathode during slurry preparation (Figure 1B). Adding a small amount of IL into the cathode slurry will only slightly change the rheological behavior of the cathode slurry, but without changing the existing battery manufacturing infrastructure. To our knowledge, few works have explored the potential of an IL-containing composite cathode for developing solid-state Li metal batteries (Feng et al., 2019; Sugata et al., 2018; Huang et al., 2021; Liu et al., 2016).

Our research has attempted to address the primary challenge of the cathode/solid electrolyte interfacial resistance in garnet-based solid-state Li-metal batteries. This paper presents an alternative strategy for the reduction of the cathode/solid electrolyte interfacial resistance by developing an IL-containing quasi-solid-state composite cathode. The introduction of an IL into the LCO cathode effectively reduced the LCO/Al-LLZO interfacial resistance. The effects of IL type and content on the electrochemical behavior of the quasi-solid-state LCO cathode were systematically studied. We demonstrated that developing an IL-containing cathode was a convenient and effective way to reduce the cathode/solid electrolyte interfacial resistance, which is also highly compatible with the existing battery manufacturing process.

## Results and discussion

### Electrochemical properties of two selected ILs and the Al-LLZO pellet

**Figure 2:**
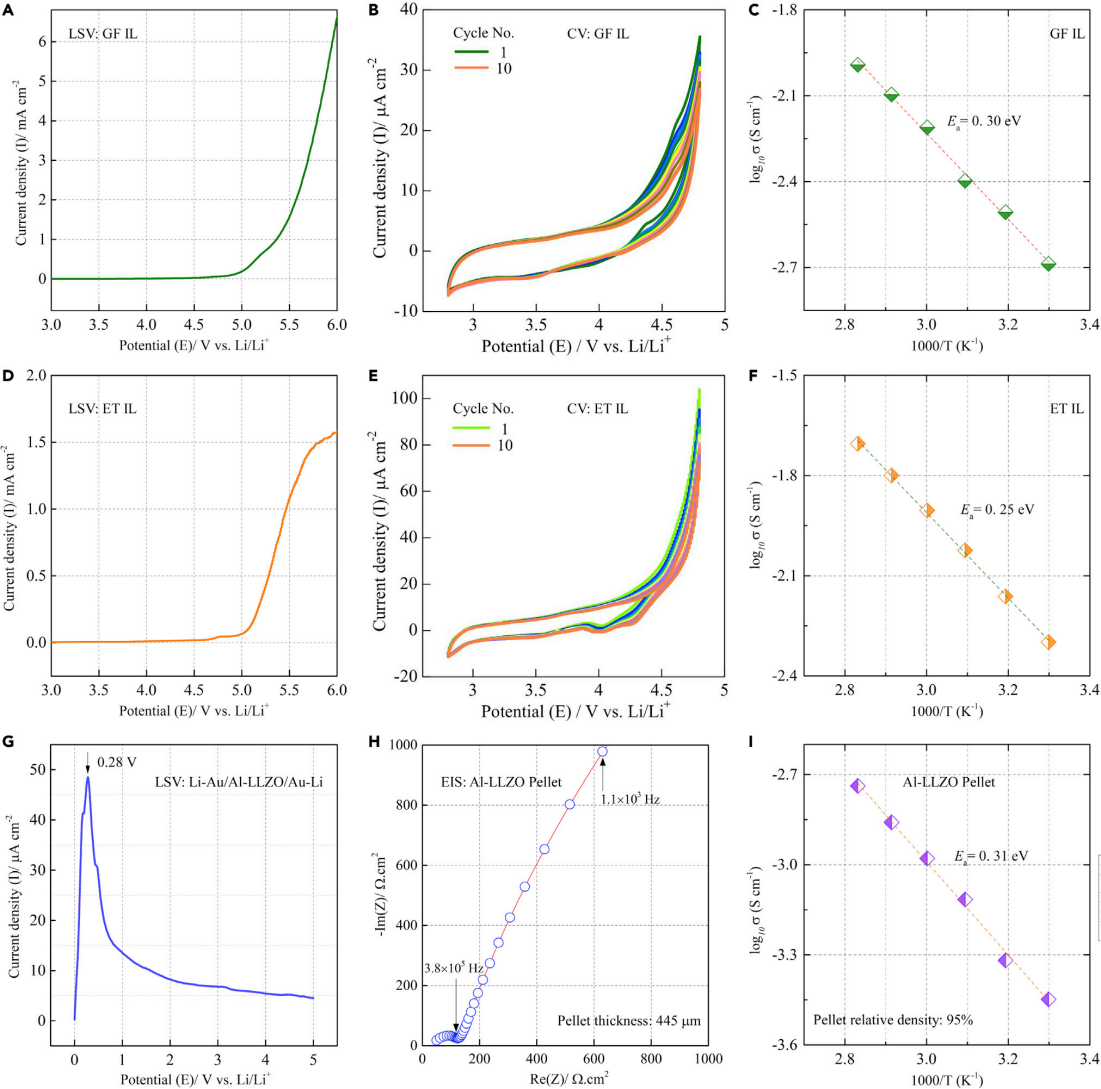
Electrochemical properties of the two selected ILs and the Al-LLZO pellet (A) Linear sweep voltammogram of GF, (B) Cyclic voltammogram of GF, (C) Arrhenius conductivity of GF, (D) Linear sweep voltammogram of ET, (E) Cyclic voltammogram of ET, (F) Arrhenius conductivity of ET. An Au foil was used as the WE, and a Li foil was used as the CE as well as the RE. A highly porous polyimide film (PI) was used as the separator. The scan rates for the LSV and CV measurements were 1 mV s^−1^, (G) Linear sweep voltammogram of a Li/Al-LLZO/Li symmetric cell at a scan rate of 1 mV min^−1^, (H) EIS spectrum of the Al-LLZO pellet at 30°C, (I) Arrhenius conductivity of the Al-LLZO pellet. All the CV and LSV measurements were carried out at 30°C.

Two types of ILs were used for developing the IL-containing quasi-solid-state composite LCO cathode. One was a solvate IL, Li(G4)FSI, designated as GF, which was prepared by dissolving lithium bis(fluorosulfonyl)imide (LiFSI) in equimolar tetraethylene glycol dimethyl ether (G4) (Cheng et al., 2020; Tsuzuki et al., 2015). Another one was a conventional IL, 1 mol dm^−3^ LiTFSI/EMI-TFSI, designated as ET, which was prepared by dissolving lithium bis(trifluoromethylsulfonyl)imide (LiTFSI) in 1-ethyl-3-methylimidazolium bis(trifluoromethylsulfonyl)imide (EMI-TFSI). As shown in Figure 2, the electrochemical stability of GF, ET, and the Al-LLZO pellet was studied by linear sweep voltammetry (LSV) and cyclic voltammetry (CV); the ionic conductivity in the temperature range of 30–80°C was measured by electrochemical impedance spectroscopy (EIS). An Au foil was used as the working electrode (WE) and a Li foil was used as the counter electrode (CE) as well as the reference electrode (RE) for the LSV and CV measurements.

GF showed good anodic stability up to 4.5 V versus Li/Li^+^ because the anodic current was almost negligible (less than 0.025 mA cm^−2^) at potentials below 4.5 V versus Li/Li^+^ (Figure 2A). The drastic increase of the current density at potentials above 5.0 V versus Li/Li^+^ indicated strong oxidation of GF. As shown in Figure 2B, no noticeable redox peaks are observed from the CV curves in the potential range of 2.8–4.8 V versus Li/Li^+^, further proving that GF has a relatively wide electrochemical window. The ionic conductivity of GF was 2.1 × 10^−3^ S cm^−1^ at 30°C and 6.2 × 10^−3^ S cm^−1^ at 60°C. In general, the ionic conductivity of a liquid electrolyte will decrease when its salt concentration is greater than 1 mol dm^−3^ because of a rapid increase in its viscosity (Yamada, 2020). It should be noted that the salt concentration in GF was as high as 4.5 mol dm^−3^. As a comparison, the ionic conductivity of the commercial liquid electrolyte, 1 mol dm^−3^ LiPF_6_/EC: DMC (v:v = 1:1), was about 1.1 × 10^−2^ S cm^−1^ at 30°C, much higher than that of GF. The temperature dependence of the ionic conductivity was fitted with the Arrhenius equation:

(Equation 1) $$\sigma \left(T\right)=\sigma_{0}\;\exp\left[-E_{a}/\left(RT\right)\right]$$

where *σ* is the ionic conductivity, *σ*_0_ is the pre-exponential factor (a constant with the same unit to *σ*), T is the absolute temperature, *E*_a_ is the activation energy and R is the universal gas constant. The activation energy of GF was estimated to be 0.3 eV (Figure 2C).

On the other hand, a noticeable anodic current increase was observed in the LSV curve of ET at potentials slightly above 4.0 V versus Li/Li^+^, and a sharp increase in the current density occurred at potentials above 5.0 V versus Li/Li^+^ (Figure 2D). In addition, broad reduction peaks were observed in the CV curves at potentials slightly above 4.0 V versus Li/Li^+^ (Figure 2E). Thus, ET has a narrower electrochemical window than GF. The ionic conductivity of ET was 5.0 × 10^−3^ S cm^−1^ at 30°C and 1.2 × 10^−2^ S cm^−1^ at 60°C. The activation energy of ET was measured to be 0.25 eV (Figure 2F), lower than that of GF. Figure 2G shows the LSV profile of a Li/Al-LLZO/Li symmetric cell in the potential range of 0–5.0 V versus Li/Li^+^. A thin Au interlayer was introduced into the Li/Al-LLZO interface to reduce the interfacial resistance. Li stripping occurred at 0.28 V versus Li/Li^+^ and no other oxidation peaks were observed up to 5.0 V versus Li/Li^+^. The ionic conductivity of the Al-LLZO pellet was 3.6 × 10^−4^ S cm^−1^ at 30°C (Figure 2H) and 1.0 × 10^−3^ S cm^−1^ at 60°C. The activation energy of the Al-LLZO pellet was 0.31 eV (Figure 2I), which was relatively high because of the low relative density of the pellet (95%). The dependence of ionic conductivity of GF, ET, and the Al-LLZO pellet as a function of temperature was presented in Tables S1–S3.

### Improvement of interfacial contact between LCO and Al-LLZO by an IL

**Figure 3:**
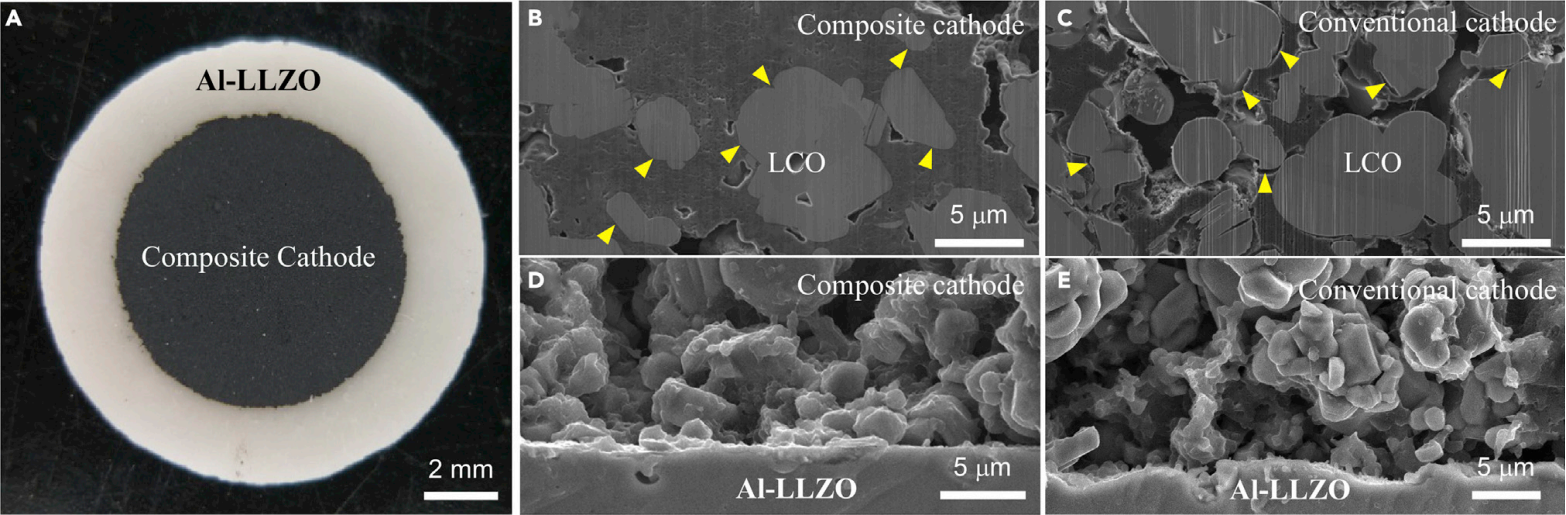
Optical and SEM images of the quasi-solid-state and a conventional LCO cathodes (A) Image of the quasi-solid-state LCO cathode, where GF was 9.1 wt%, (B) Cross-sectional SEM micrograph of the quasi-solid-state LCO cathode, (C) Cross-sectional SEM micrograph of a conventional LCO cathode (without IL), (D) SEM micrograph of the interface region between the quasi-solid-state LCO cathode and the Al-LLZO pellet, (E) SEM micrograph of the interface region between the conventional LCO cathode and the Al-LLZO pellet. All the cross-sections were prepared by focused ion beam (FIB) milling.

A conventional LCO cathode slurry was prepared by mixing an LCO powder, an AB conductive agent, and a PVDF powder in NMP. Either GF or ET was directly added into the conventional LCO slurry to make an IL-containing composite LCO slurry. Figure 3A shows an optical image of the IL-containing composite LCO cathode, which was directly cast onto the Al-LLZO pellet. The experimental procedure is shown in Figure S1. The composite LCO cathode is quasi-solid and shows no fluidity. A cross-sectional SEM micrograph of the composite cathode is shown in Figure 3B, where LCO particles are firmly embedded in a quasi-solid-state matrix, consisting of GF, AB, and PVDF. In contrast, voids and cracks are observed at the LCO/matrix interface region in a conventional LCO cathode, as indicated by the arrowheads in Figure 3C. Figure 3D presents a cross-sectional SEM micrograph of the interface region between the quasi-solid-state LCO cathode and the Al-LLZO pellet. The LCO cathode layer was about 30 μm in thickness and formed intimate contact with the Al-LLZO pellet. Figure 3E presents a cross-sectional SEM micrograph of the interface region between the conventional LCO cathode and the Al-LLZO pellet, where more voids are observed. Thus, the introduction of an IL could not only reduce the interfacial resistance between LCO and Al-LLZO but could also reduce the internal resistance of the LCO cathode. Moreover, an interaction between GF and LCO was proved by differential scanning calorimetry (DSC) (Figure S2).

### Cycling performance of the GF-containing LCO cathode

**Figure 4:**
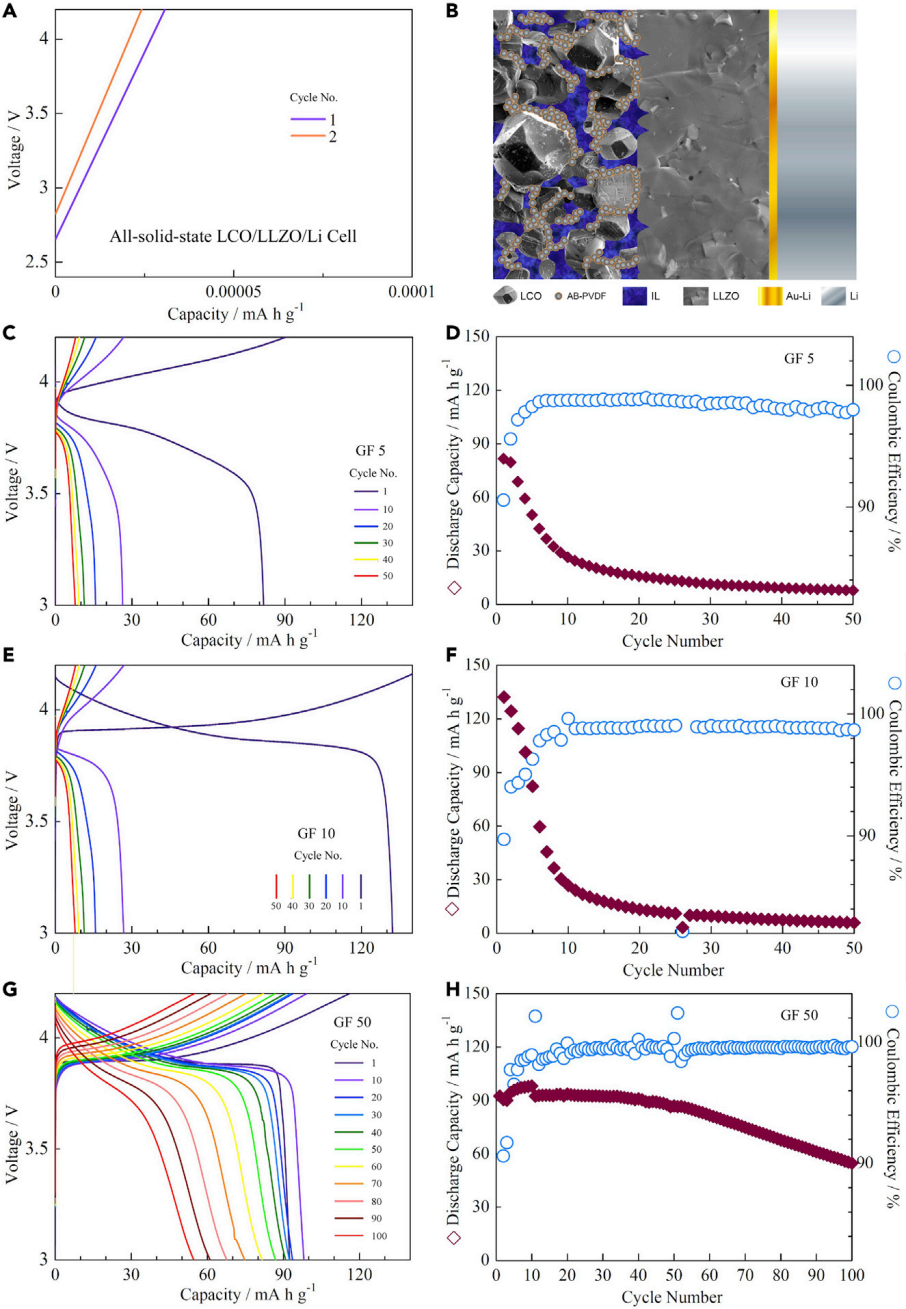
Electrochemical behavior of quasi-solid-state LCO/Al-LLZO/Li cells with different GF contents (A–H) (A) *x* = 0 (all-solid-state), (B) Illustration of the configuration of the quasi-solid-state LCO/Al-LLZO/Li cell, (C and D) Electrochemical behavior of cell GF 5 (*x* = 5), (E and F) Electrochemical behavior of cell GF 10 (*x* = 10), and (G and H) Electrochemical behavior of cell GF 50 (*x* = 50). The *C*-rate is 0.025C and the cycling temperature is 60°C.

The weight ratio of LCO: AB: PVDF: GF in the GF-containing LCO composite cathode was 92: 4: 4: *x*, where *x* = 0, 5, 10, 20, or 50. The assembled coin cells were termed GF-0, GF-5, GF-10, GF-20, and GF-50, accordingly. The IL contents in the quasi-solid-state LCO cathodes in terms of both weight and volume percentages are listed in Table S4. All-solid-state LCO/Al-LLZO/Li batteries cannot be reversibly charged and discharged, whereas quasi-solid-state LCO/Al-LLZO/Li batteries can. As shown in Figure 4A, the all-solid-state LCO/Al-LLZO/Li cell (*x* = 0) reaches the cut-off voltage of 4.2 V in seconds and no discharge occurs. This was very likely due to the large interfacial resistance between the conventional LCO cathode and the Al-LLZO pellet, because the anode-side interfacial resistance between Al-LLZO and Li could be reduced to as low as a few Ω cm^2^ by introducing a thin Au interlayer or by simply removing the common LLZO surface contaminants, such as Li_2_CO_3_ (Wakasugi et al., 2017; Inada et al., 2018; Sharafi et al., 2017; Yamada et al., 2020). To reduce the cathode-side interfacial resistance, the concept of an IL-containing quasi-solid-state cathode is thus explored and the cell configuration is illustrated in Figure 4B. The GF-containing (*x* = 5) quasi-solid-state LCO cathode can be reversibly charged and discharged (Figure 4C). The initial discharge capacity was 81.6 mA h g^−1^, which decreased rapidly with cycling. Although the Coulombic efficiency (CE) increased upon cycling (greater than 98% after the fifth cycle), the discharge capacity was only 7.8 mA h g^−1^ after 50 cycles (Figure 4D). The initial discharge capacity increased to 132.2 mA h g^−1^ at *x* = 10 (Figure 4E), which was likely because of a further reduced interfacial resistance at the cathode side. Similar to the case of *x* = 5, although the average CE was greater than 98%, the discharge capacity decreased quickly and was only 5.9 mA h g^−1^ after 50 cycles (Figure 4F). At *x* = 50, the cyclability increased significantly (Figure 4G) and an average CE greater than 99% was maintained after 100 cycles (Figure 4H). However, it should be noted that the initial discharge capacity was relatively low (92.4 mA h g^−1^) and it degraded rapidly after 50 cycles. This was probably due to the thermal instability of GF because it was electrochemically stable up to 4.5 V versus Li/Li^+^. Besides, although the solid-state LCO/Al-LLZO interface could be fully wetted by excessive IL, electron transport would be blocked to some extent, because the ILs were usually electrically insulating. In addition, particle agglomeration was also likely to occur, which further reduced the utilization of LCO.

### Composition analysis of GF after prolonged cycling at 60°C

**Figure 5:**
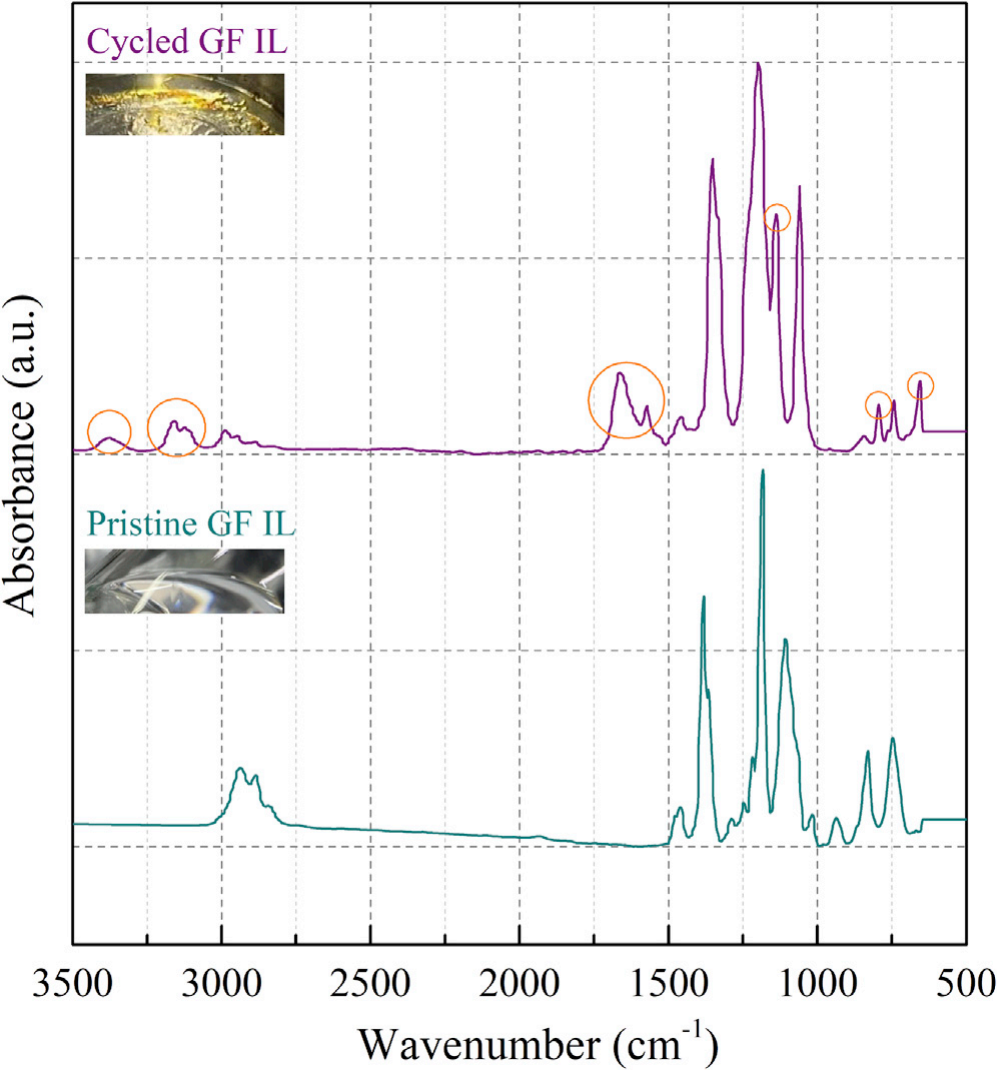
FTIR analysis of the composition change of GF after cycling The insets are optical images of GF before (transparent) and after (yellowish) prolonged cycling at 60°C.

One major reason for the degradation of GF cells was likely because of the thermal instability of GF. This was evidenced by the discoloration of GF after prolonged cycling at 60°C. Pristine GF was a transparent liquid; however, it changed to a yellowish gel after cycling (insets in Figure 5). Fourier transform infrared spectroscopy (FTIR) was used to analyze the composition change of GF after cycling at 60°C.

Almost all the vibrational modes identified in the transmission FTIR spectrum of pristine GF are present in the spectrum of cycled GF (Figure 5). For example, the strongest band at around 1185 cm^−1^ is assigned to the stretching mode of the SO_2_ unit. In addition, some new features were observed, which were likely related to the decomposition products of GF. The additional bands at about 3350 and 1600 cm^−1^ were assigned to water, which was probably introduced during cathode preparation. The additional bands at about 3140, 1140, 790, and 660 cm^−1^ were assigned to CH, SO_2_, SF, and SNS units, respectively (Kerner et al., 2016). Because GF was electrochemically stable up to 4.5 V versus Li/Li^+^ (Figure 2A), it was probably thermally decomposed during the prolonged cycling at 60°C. The thermal decomposition temperature of LiFSI (salt in GF) was 66.9°C (Kubota et al., 2008), only slightly higher than the cell cycling temperature. As a result, the cell internal temperature could exceed the thermal decomposition temperature of LiFSI during the prolonged cycling test because of heat accumulation.

### Cycling performance of the ET-containing LCO cathode

**Figure 6:**
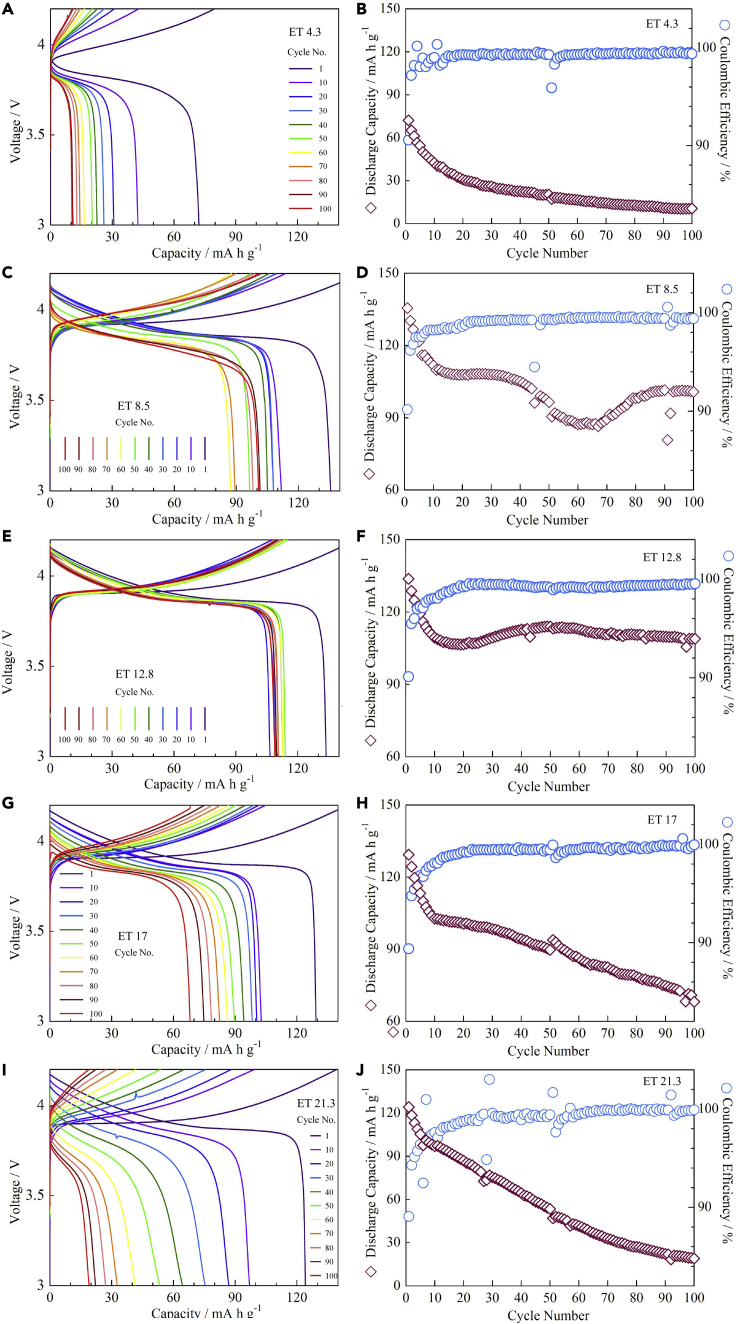
Electrochemical behavior of quasi-solid-state LCO/Al-LLZO/Li cells with different ET contents (A–J) (A and B) ET 4.3 (*x* = 4.3), (C and D) ET 8.5 (*x* = 8.5), (E and F) ET 12.8 (*x* = 12.8), (G and H) ET 17 (*x* = 17), and (I and J) ET 21.3 (*x* = 21.3). The *C*-rate is 0.025C and the cycling temperature is 60°C.

The performance of the quasi-solid-state LCO cathode was not only dependent on the IL content but also dependent on the IL type. Figure 6 shows the galvanostatic cycling results of the ET-containing quasi-solid-state LCO/Al-LLZO/Li cells. The weight ratio of LCO: AB: PVDF: ET in the ET-containing LCO cathode was also 92: 4: 4: *x*, while where *x* = 4.3, 8.5, 12.8, 17, or 21.3. Similarly, the assembled coin cells were termed ET-4.3, ET-8.5, ET-12.8, ET-17, and ET-21.3, accordingly. When *x* = 4.3, the initial discharge capacity was 72.1 mA h g^−1^, which decreased rapidly to 30.8 mA h g^−1^ after 20 cycles, and was only 10.5 mA h g^−1^ after 100 cycles (Figure 6A). However, a stable CE of about 100% was maintained after 100 cycles (Figure 6B). The discharge capacity improved significantly with increasing IL content. When *x* = 8.5, the initial discharge capacity reached 135.5 mA h g^−1^. However, it decreased quickly to 111.7 mA h g^−1^ after the first 10 cycles and was 100.7 mA h g^−1^ after 100 cycles (Figure 6C). A high CE of about 100% for over 100 cycles was also achieved at *x* = 8.5 (Figure 6D). The cyclability was further improved at *x* = 12.8 (Figure 6E). The discharge capacity was greater than 108.9 mA h g^−1^ after 100 cycles and the CE increased upon cycling and reached about 100% after 20 cycles (Figure 6F). In addition, the overpotential of the quasi-solid-state LCO/Al-LLZO/Li cell decreased with increasing IL content when *x* ≤ 12.8. Further increasing the IL content resulted in a decrease in the cell performance. As shown in Figure 6G (*x* = 17), the discharge capacity decreased continuously from an initial value of 129.3 to 68.1 mA h g^−1^ after 100 cycles, although the CE was reached about 100% after 20 cycles (Figure 6H).

The adverse effect of excessive IL became more apparent when *x* = 21.3 (Figure 6I). The discharge capacity decreased rapidly from an initial value of 124.1 mA h g^−1^ to only 19.0 mA h g^−1^ after 100 cycles. Significant fluctuations of the CE were also observed (Figure 6J). Moreover, the cell overpotential increased with increasing IL content when *x* ≥ 12.8. As discussed above for the case of GF, excessive IL could block electron transport and cause particle agglomeration in the quasi-solid-state LCO cathode. Nonetheless, the ET-containing LCO cathode showed a much better cycling performance than the GF-containing LCO cathode, probably because ET had both higher thermal stability and higher ionic conductivity than GF.

### EIS analysis of the quasi-solid-state LCO/Al-LLZO/Li cell

**Figure 7:**
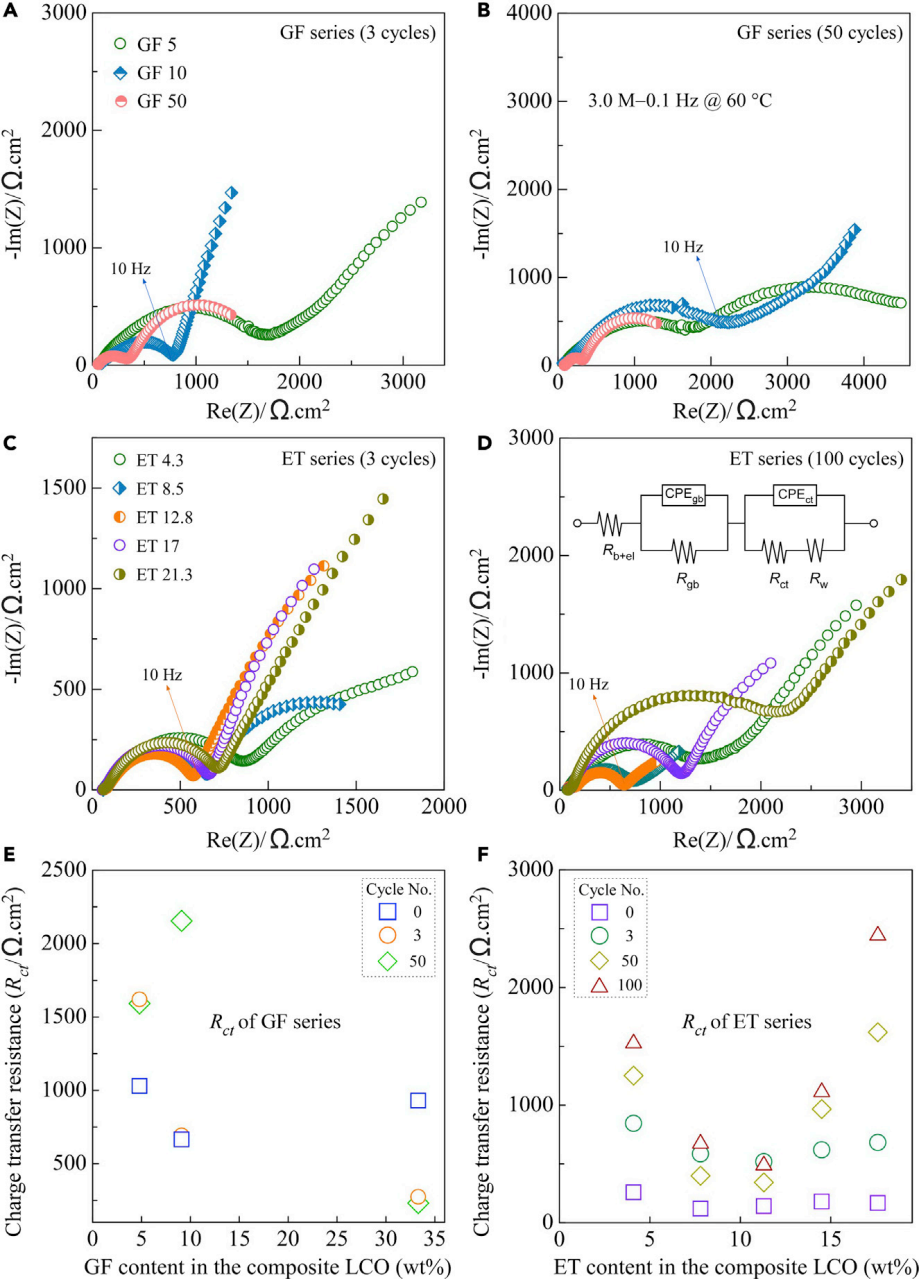
Impedance evolution of quasi-solid-state LCO/Al-LLZO/Li cells at 60°C as functions of IL type, IL content, and cycle number (A) EIS spectra of the GF-containing LCO/Al-LLZO/Li cell after three cycles, (B) EIS spectra of the GF-containing LCO/Al-LLZO/Li cell after 50 cycles, (C) EIS spectra of the ET-containing LCO/Al-LLZO/Li cell after three cycles, (D) EIS spectra of the ET-containing LCO/Al-LLZO/Li cell after 50 cycles, (E) *R*_ct_ of the GF-containing LCO/Al-LLZO/Li cell and (F) *R*_ct_ of the ET-containing LCO/Al-LLZO/Li cell.

To better understand the effect of the IL on the performance of the quasi-solid-state LCO/Al-LLZO/Li cell, EIS analysis was carried out (Figure 7). Each spectrum generally consists of two semicircles, a smaller one in the high-frequency region (≥1 MHz) (not readily visible), corresponding to the resistance of Al-LLZO (*R*_LLZO,_ including both the grain boundary resistance *R*_gb_ and the bulk resistance *R*_b_), and a larger one in the low-frequency region (<1 MHz), corresponding to the charge transfer resistance *R*_ct_. For the GF-containing LCO/Al-LLZO/Li cells, the overall resistance (*R*_overall_) decreased significantly from about 1660 Ω cm^2^ at *x* = 5 to about 320 Ω cm^2^ at *x* = 50 (Figure 7A). The area-specific resistance (ASR) was defined as,

ASR = A×R

where *A* is the effective current collecting area and *R* is the measured resistance. As a comparison, the *R*_overall_ of the all-solid-state LCO/Al-LLZO/Li cell was measured to be higher than 500,000 Ω cm^2^. Considering that the anode-side interfacial resistance between Al-LLZO and Li was only about a few to several hundred Ω cm^2^ (Tsai et al., 2016; Inada et al., 2018; Sharafi et al., 2017; Taylor et al., 2018), the *R*_ct_ was therefore dominated by the cathode-side interfacial resistance between LCO and Al-LLZO. After 50 cycles, a substantial increase in *R*_overall_ from about 750 to 2230 Ω cm^2^ was observed for *x* = 10. In comparison, there was almost no change in *R*_overall_ for *x* = 5 and *x* = 50 (Figures 7A and 7B), probably because GF was either too little (*x* = 5) or too much (*x* = 50) in the composite LCO cathode.

The EIS spectra of the ET-containing LCO/Al-LLZO/Li cells are shown in Figures 7C and 7D. The *R*_overall_ was about 830 Ω cm^2^ at *x* = 4.3, which decreased to about 560 Ω cm^2^ at *x* = 12.8. However, the *R*_overall_ increased slightly with further increasing the ET content (Figure 7C). After 100 cycles, the *R*_overall_ increased noticeably for all cells (Figure 7D). The evolution of the *R*_overall_ was correlated well with the cell performance (Figure 6). The EIS data were analogized with a simplified equivalent circuit (inset in Figure 7D), where *R*_ct_ represented the sum of the interfacial resistance from both the cathode and anode sides.

As plotted in Figures 7E and 7F, *R*_ct_ of the GF cell is in the range of 600–1000 Ω cm^2^ before cycling, while that of the ET cells is in the range of 120–250 Ω cm^2^. After cycling, the *R*_ct_ increased significantly in almost all the cases, irrespective of the IL type and content, indicating that decomposition of the ILs or interfacial side reactions should have occurred. Nevertheless, ET was shown to be more suitable than GF for preparing the IL-containing quasi-solid-state LCO cathode. As a result, more detailed research was conducted on the stability analysis of the Al-LLZO pellet and the LCO active material against ET.

### Stability study of Al-LLZO against ET by XPS

**Figure 8:**
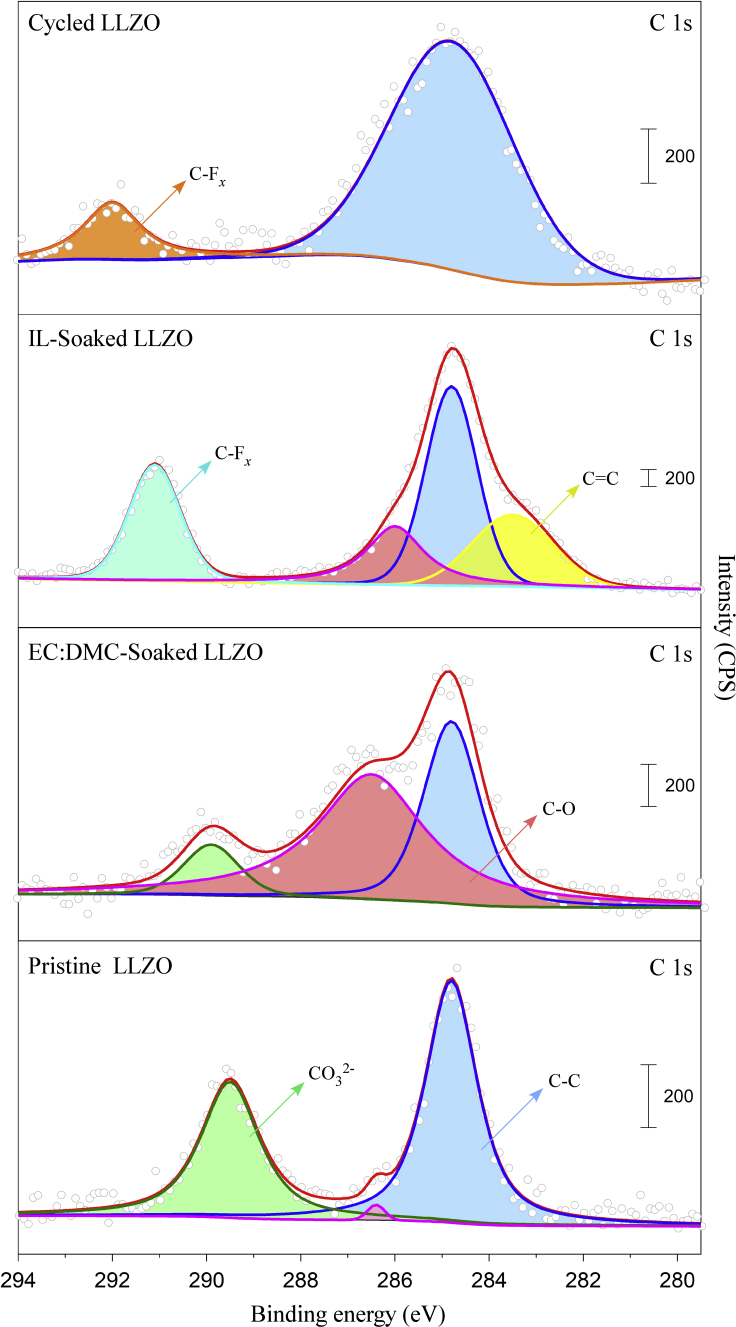
XPS analysis of the surface chemistry (C 1s) of the Al-LLZO pellet in different conditions As-polished (pristine), soaked in 1 mol dm^−3^ LiPF_6_/EC-DMC (v:v = 1:1) (LP30) for 200 h, soaked in ET for 200 h, and galvanostatically cycled for 100 cycles at 60°C.

Because the IL-containing LCO cathode/Al-LLZO pellet interface could be divided into two distinct solid/liquid interfaces, i.e., LCO/IL and IL/Al-LLZO, the stability of the cathode-side interfaces was thus dependent on the stability of LCO and Al-LLZO against the IL. Figure 8 shows the X-ray photoelectron spectroscopy (XPS) analysis results of Al-LLZO in different conditions: as-polished (pristine), soaked in a 1 mol dm^−3^ LiPF_6_/EC: DMC (v: v = 1:1) (LP30) electrolyte for 200 h, soaked in ET for 200 h, and galvanostatically cycled for 100 cycles at 60°C. Adventitious carbon (centered at 248.8 eV in C 1s) and carbonate (289. 7 eV in C 1s) were observed on the surface of the Al-LLZO pellet which was polished in an Ar-filled glove box. As reported previously, surface contaminants, such as Li_2_CO_3_, would occur even in an Ar atmosphere (Yamada et al., 2020). Apparent enrichment in C-O species (286.5 eV in C 1s) indicated that a reaction between Al-LLZO and LP30 occurred, which was also observed by Liu et al. (2020). On the other hand, almost no carbonate signal was observed in both the C 1s and O 1s spectra (Figure S3) of the ET-soaked Al-LLZO pellet, and the amount of the C-O species was relatively low, suggesting that Al-LLZO was relatively stable against ET. However, the presence of carbon (248.8 eV in C 1s) on the Al-LLZO surface increased significantly after 100 cycles at 60°C, probably because of the electrochemical decomposition of ET or reactions between Al-LLZO and ET.

### Stability study of LCO against ET by TEM

**Figure 9:**
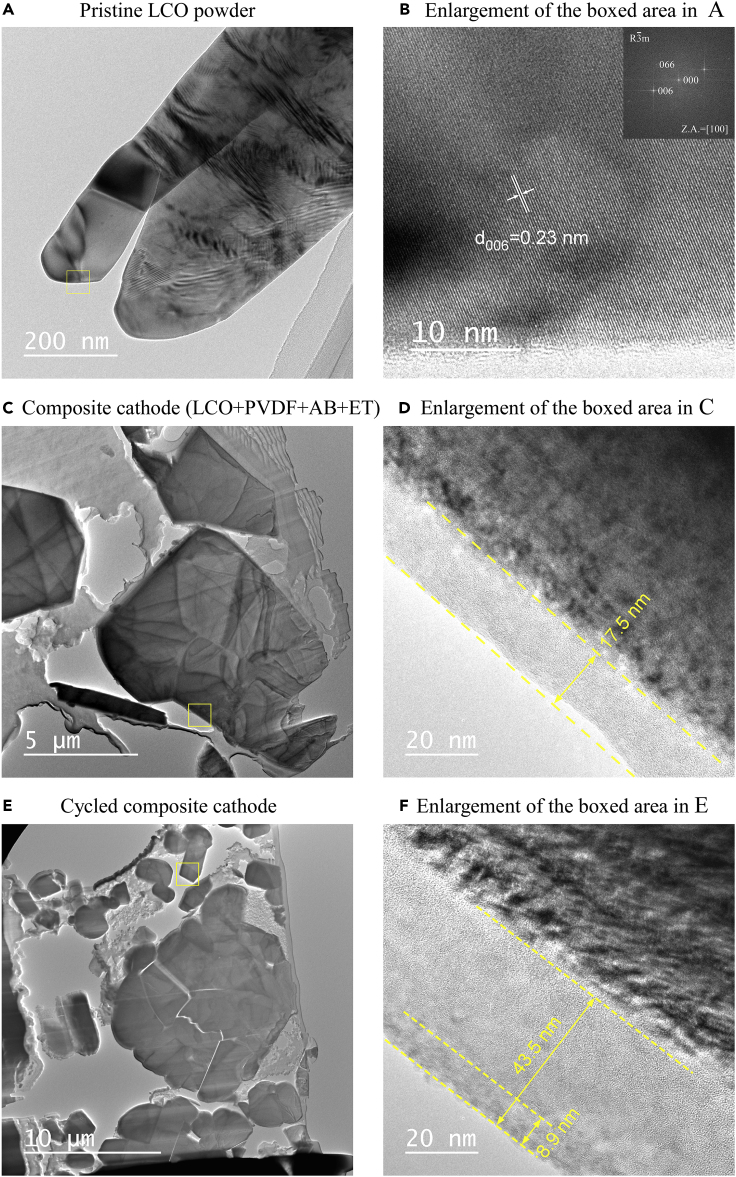
Bright-field TEM micrographs of the LCO active material (A–F) (A and B) Pristine LCO powder, (C and D) ET-containing quasi-solid-state LCO cathode before cycling, (E and F) ET-containing quasi-solid-state LCO cathode after 100 cycles at 60°C. The inset in (B) is a fast Fourier transform (FFT) pattern of the high-resolution TEM image

The stability of LCO against ET was investigated using TEM. As shown in Figure 9A, the pristine LCO particle has a crystalline appearance, which is revealed by the high-resolution TEM image shown in Figure 9B. The well-defined layered structure with an interplanar spacing of about 0.23 nm is indexed to be the (006) crystal plane of LCO. No additional or amorphous surface layer is observed. A TEM micrograph of the quasi-solid-state composite LCO cathode before cycling is shown in Figure 9C, where the LCO particles are embedded in a PVDF polymer matrix with a brighter contrast. An enlargement of the boxed area without PVDF is shown in Figure 9D, where an amorphous cathode electrolyte interphase (CEI) layer about 17.5 nm in thickness is observed, suggesting that reactions occurred at the IL/LCO interface. Cracking of cycled LCO particles was observed (Figure 9E), indicating that stress build-up in the quasi-solid-state battery was severe during prolonged cycling. A high-resolution TEM image of the boxed area without any PVDF is shown in Figure 9F, where the thickness of the CEI layer has increased significantly from about 17.5 to 43.5 nm. The continuous growth of the CEI layer would lead to the increase of *R*_ct_. In addition, the outermost part of the CEI layer (about 8.9 nm thick) with a darker contrast showed a more crystalline structure, which was likely related to the transfer of heavy elements, such as Co.

### Components of the CEI layer analyzed by XPS

**Figure 10:**
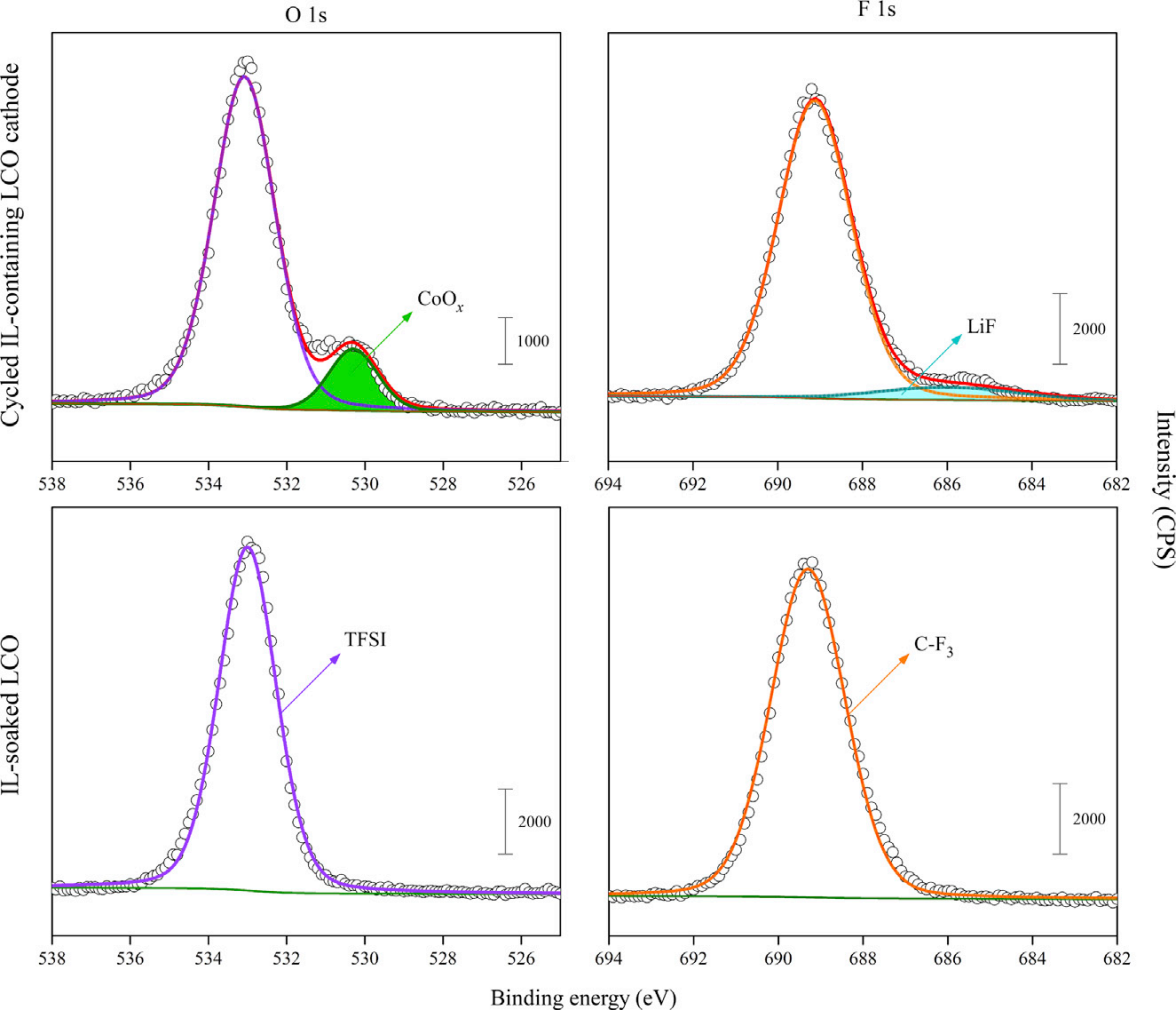
XPS analysis of the components of the CEI layer formed on LCO surface in different conditions Soaked in ET for 200 h and Cycled at 60°C for 100 cycles.

To understand the reasons for the continuous growth of the CEI layer formed on the LCO surface, XPS analysis was carried out. As shown in Figure 10, the appearance of the peak assigned to CoO_*x*_ at 530.3 eV suggests the decomposition of LCO (Dahéron et al., 2008). The appearance of the peak assigned to the Li-F bond at 686.0 eV suggests the decomposition of the LiTFSI salt in ET (Jafta et al., 2019). In addition, carbonaceous species, such as C-C and C-H, were identified in the CEI layer. Thus, the CEI layer on LCO consisted of both inorganic and organic compounds that were likely the decomposition products of LCO and ET.

### Failure mechanisms of the quasi-solid-state LCO/Al-LLZO/Li cell

**Figure 11:**
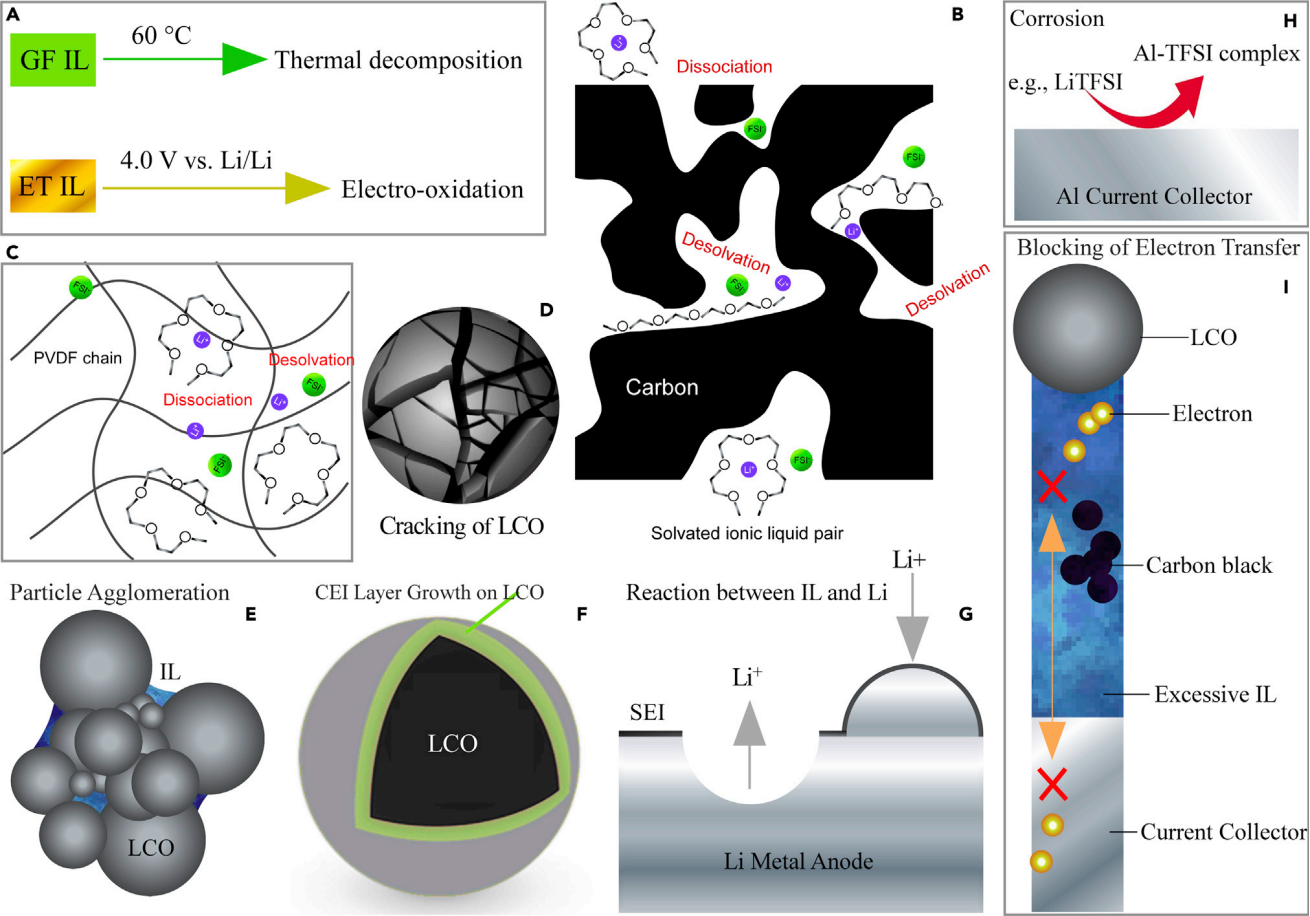
Illustration of failure mechanisms of the quasi-solid-state LCO cathode (A–I) (A) Thermal decomposition or electron-oxidation of the ILs, (B and C) Dissociation or desolvation of GF in carbon black and PVDF binder, (D) Cracking of LCO caused by stress accumulation and fatigue, (E) Agglomeration of LCO (or AB) particles, (F) Growth of the CEI layer on LCO because of reactions between LCO and the IL, (G) Possible reaction between Li metal and the ILs, (H) Corrosion of Al current collector in the ILs and (I) Blocking of electron transport by excessive IL.

Failure mechanisms of the quasi-solid-state LCO/Al-LLZO/Li cell are illustrated in Figure 11. First of all, GF was thermally decomposed after prolonged cycling at 60°C, as indicated by the FTIR analysis, although it was electrochemically stable up to 4.5 V versus Li/Li^+^. On the other hand, ET became electrochemically oxidized at potentials slightly above 4.0 V versus Li/Li^+^, as evidenced by the LSV and CV analysis. The instabilities of the ILs were considered to be the main reasons for the decay of the quasi-solid-state Li metal batteries. In addition, GF might dissociate or desolvate because of the nanoconfinement effects from the nanoporous carbon black and the chain-like PVDF binder (Figures 11B and 11C) (Borghi et al., 2021; Cheng et al., 2022). Because the interaction between the tetraglyme and Li^+^ ion was relatively weak, the chelate complex was intrinsically unstable (Mandai et al., 2014). Cracking of LCO particles was revealed by TEM analysis, which was likely because of stress accumulation in the quasi-solid-state battery and fatigue of LCO during prolonged cycling (Figure 11D). As illustrated in Figure 11E, particle agglomeration in ET was also confirmed by dynamic light scattering (DLS) analysis (Figure S4). This decreased the utilization of the cathode active material. The continuous growth of the CEI layer on LCO was another reason for the gradual decay of the cell capacity (Figure 11F). The EIS, XPS, and TEM analysis confirmed the growth of the CEI layer with cycling, which was likely related to the reactions between ET and LCO. Meanwhile, the reaction between LLZO and ET was indicated by XPS analysis. Possible reactions between Li metal and the ILs could also be one of the reasons for the capacity decay of the IL-containing LCO/Al-LLZO/Li battery (Figure 11G). Similarly, reactions between the ILs and the Al current collector, i.e., corrosion (Ma et al., 2017), would occur (Figure 11H). Broad redox peaks were observed in the CV profiles of GF when an Al foil was used as the working electrode (Figure S5), likely because of the corrosion of Al in GF. The charge transfer pathway between LCO and the Al current collector would be blocked to some extent when the IL was excessive, because the electron-conductive carbon black particles could be isolated by the excessive IL, which was electronically insulating (Figure 11I).

Despite the shortcomings of the quasi-solid-state composite LCO cathode, there are strategies to improve its performance. First, the search for a more suitable IL is essential. Second, coating the cathode active material and the solid electrolyte with a Li-ion conductive material, such as LiNbO_3_ or LiTaO_3_, can be a feasible way to improve their stability in the ILs (Wang et al., 2020b).

## Conclusion

This paper presents an alternative strategy for reducing the interfacial resistance issue between cathodes and solid electrolytes by developing an IL-containing composite cathode. An IL-containing and nonfluidic LCO composite cathode was developed and the LCO/Al-LLZO interfacial resistance was reduced significantly from more than 500,000 to about 100 Ω cm^2^. The optimal content of the IL in the quasi-solid-state composite LCO cathode was found to be about 11 wt%. Quasi-solid-state LCO/Al-LLZO/Li cells achieved relatively high capacity retention of about 80% after 100 cycles at 60°C. The cell capacity decay was mainly because of the thermal or electrochemical instability of the IL. There were also stability issues of LCO and Al-LLZO against the IL. Thus, the search for a suitable IL is critical for the further development and maturation of the garnet-based quasi-solid-state Li metal batteries.

### Limitations of the study

The two ionic liquids (ILs) used in this study, i.e., the solvate equimolar Li(G4)FSI and the conventional 1 mol dm^−3^ LiTFSI/EMI-TFSI, are either thermally unstable above 60°C or become electrochemically oxidized at potentials above 4.0 V versus Li/Li^+^. In addition, it should be noted that the viscosities of the two ILs at room temperature are relatively high. Thus, identifying a suitable IL is critical for the further development of the quasi-solid-state composite LiCoO_2_ cathode. In addition, the relative density of the Al-doped LLZO pellet is relatively low, about 95%, because it was prepared by pressureless sintering. Better battery performance is expected if a denser or a Ta-doped LLZO pellet (with a higher ionic conductivity) is used.

## STAR★Methods

### Key resources table

**Table:** 

REAGENT or RESOURCE	SOURCE	IDENTIFIER
**Chemicals and reagents**		

Zirconium dioxide	Tosoh Corp.	CAS# 1314-23-4
Gamma alumina	Kojundo Chemical Laboratory Co., Ltd.	CAS# 1344-28-1
Lithium hydroxide monohydrate	Kojundo Chemical Laboratory Co., Ltd.	CAS# 1310-66-3
Lanthanum hydroxide	Kojundo Chemical Laboratory Co., Ltd.	CAS#1312-81-8
Lithium cobalt oxide	MTI Materials Pvt. Ltd	CAS#12190-79-3
Lithium foil	Honjo Metal Co., Ltd	CAS# 7439-93-2
Tetraethylene glycol dimethyl ether	Kishida Chemical Co., Ltd.	CAS# 143-24-8
1-ethyl-3-methylimidazolium bis(trifluoromethanesulfonyl)imide	Kishida Chemical Co., Ltd.	CAS# 174899-82-2
Lithium bis(fluorosulfonyl)imide	Kishida Chemical Co., Ltd.	CAS# 171611-11-3
Lithium bis(trifluoroethanesulfonyl)imide	Kishida Chemical Co., Ltd.	CAS# 90076-65-6

### Resource availability

#### Lead contact

Further information and requests for resources and reagents should be directed to and will be fulfilled by the lead contact, Kiyoshi Kanamura (kanamura@tmu.ac.jp).

#### Materials availability

No new material was generated in this study.

### Method details

#### Preparation of Al-doped LLZO pellets

Al-doped LLZO powder was prepared by a solid-state reaction method (Kotobuki et al., 2011). LiOH·H_2_O, La(OH)_3_ and ZrO_2_ powders were mixed by planetary ball-milling and were calcined at 900°C for 15 h. After mixing with a γ-Al_2_O_3_ powder, the calcined powder was pelletized and sintered at 900°C for 3 h and then at 1200°C for 24 h. The molar ratio of the starting materials of LiOH·H_2_O, La(OH)_3_, ZrO_2,_ and γ-Al_2_O_3_ was 6.9: 3.0 : 2.0: 0.125.

#### Fabrication of quasi-solid-state LCO/Al-LLZO/Li coin cells

The IL-containing LCO slurries were cast onto the cylinder-shaped Al-LLZO pellets (12 mm in diameter and 1 mm in thickness) and then dried at 80°C in vacuum for 12 h. The loading of the active material was about 6.0 mg cm^−2^. Another base of the pellet was coated with a thin gold layer, which would alloy with Li metal at heating (80°C) to reduce the anode-side interfacial resistance. The quasi-solid-state LCO/Al-LLZO/Au trilayered structure was paired with a Li foil to make a full cell. All the CR2032-type coin cells were assembled in an Ar-filled glove box.

#### Materials characterization

The crystal structure of the high-temperature sintered Al-LLZO pellet was analyzed by X-ray diffraction (XRD, Rigaku SmartLab) (Figure S6). Micro-sized pores were observed on the cross-sectional surface of the Al-LLZO pellet (Figure S7). The relative density of the Al-LLZO pellet was about 95%. Cross-sections of the Al-LLZO/LCO bilayer structure were prepared with focused ion beam (FIB) milling and were analyzed using a scanning electron microscope (SEM, JSM-6490A). The particle size in the LCO slurry was measured by dynamic light scattering (DLS, Partica mini LA-350). Morphological evolution of the LCO surface was studied using an analytical scanning transmission electron microscope (STEM, JEM-ARM200F). Transmission and attenuated total reflection (ATR) Fourier transform infrared spectroscopy (FTIR) was used to analyze the composition change of the IL after cycling at 60°C. The surface chemistry of Al-LLZO was analyzed with an X-ray photoelectron spectrometer (XPS, PHI 5000 VersaProbe II).

#### Electrochemical measurements

AC electrochemical impedance spectroscopy was performed in the frequency range from 3 MHz to 0.1 Hz at 60°C (EIS, Biologic SP-300). The amplitude of the perturbation was 10 mV. Galvanostatic cycling of the quasi-solid-state LCO/Al-LLZO/Li cell was carried out at 60°C with a constant current density of 20 μA cm^−2^, corresponding to 1/40C (0.025C). The cut-off voltage range was 3.0–4.2 V.

## Data Availability

• All data reported in this paper will be shared by the lead contact upon request. • No custom code was used. This is an experimental study of garnet-based quasi-solid-state Li metal batteries. • Any additional information required to reanalyze the data reported in this paper is available from the lead contact upon request.

## References

[bib1] Borghi F., Piazzoni C., Ghidelli M., Milani P., Podestà A. (2021). Nanoconfinement of ionic liquid into porous carbon electrodes. J. Phys. Chem. C.

[bib2] Cheng E.J., Kimura T., Shoji M., Ueda H., Munakata H., Kanamura K. (2020). Ceramic-based flexible sheet electrolyte for Li batteries. ACS Appl. Mater. Inter..

[bib3] Cheng E.J., Liu M., Li Y., Abe T., Kanamura K. (2022). Effects of porosity and ionic liquid impregnation on ionic conductivity of garnet-based flexible sheet electrolytes. J. Power Sources.

[bib4] Cheng E.J., Taylor N.J., Wolfenstine J., Sakamoto J. (2017). Elastic properties of lithium cobalt oxide (LiCoO_2_). J. Asian Ceram. Soc..

[bib5] Cui G. (2020). Reasonable design of high-energy-density solid-state lithium-metal batteries. Matter.

[bib6] Dahéron L., Dedryvère R., Martinez H., Ménétrier M., Denage C., Delmas C., Gonbeau D. (2008). Electron transfer mechanisms upon lithium deintercalation from LiCoO_2_ to CoO_2_ investigated by XPS. Chem. Mater..

[bib7] Famprikis T., Canepa P., Dawson J.A., Islam M.S., Masquelier C. (2019). Fundamentals of inorganic solid-state electrolytes for batteries. Nat. Mater..

[bib8] Fang C., Wang X., Meng Y.S. (2019). Key issues hindering a practical lithium-metal anode. Trends Chem..

[bib9] Feng W., Dong X., Li P., Wang Y., Xia Y. (2019). Interfacial modification of Li/Garnet electrolyte by a lithiophilic and breathing interlayer. J. Power Sources.

[bib10] Han X., Gong Y., Fu K.K., He X., Hitz G.T., Dai J., Pearse A., Liu B., Wang H., Rubloff G. (2017). Negating interfacial impedance in garnet-based solid-state Li metal batteries. Nat. Mater..

[bib11] Huang W., Bi Z., Zhao N., Sun Q., Guo X. (2021). Chemical interface engineering of solid garnet batteries for long-life and high-rate performance. Chem. Eng. J..

[bib12] Inada R., Yasuda S., Hosokawa H., Saito M., Tojo T., Sakurai Y. (2018). Formation and stability of interface between garnet-type Ta-doped Li_7_La_3_Zr_2_O_12_ solid electrolyte and lithium metal electrode. Batteries.

[bib13] Jafta C.J., Sun X.G., Veith G.M., Jensen G.V., Mahurin S.M., Paranthaman M.P., Dai S., Bridges C.A. (2019). Probing microstructure and electrolyte concentration dependent cell chemistry via operando small angle neutron scattering. Energy Environ. Sci..

[bib14] Kerner M., Plylahan N., Scheers J., Johansson P. (2016). Thermal stability and decomposition of lithium bis (fluorosulfonyl) imide (LiFSI) salts. RSC Adv..

[bib15] Kim H.W., Manikandan P., Lim Y.J., Kim J.H., Nam S.C., Kim Y. (2016). Hybrid solid electrolyte with the combination of Li_7_La_3_Zr_2_O_12_ ceramic and ionic liquid for high voltage pseudo-solid-state Li-ion batteries. J. Mater. Chem. A.

[bib16] Kim K.J., Balaish M., Wadaguchi M., Kong L., Rupp J.L. (2021). Solid-state Li–metal batteries: challenges and horizons of oxide and sulfide solid electrolytes and their interfaces. Adv. Energy Mater..

[bib17] Kotobuki M., Kanamura K., Sato Y., Yoshida T. (2011). Fabrication of all-solid-state lithium battery with lithium metal anode using Al_2_O_3_-added Li_7_La_3_Zr_2_O_12_ solid electrolyte. J. Power Sources.

[bib18] Krauskopf T., Richter F.H., Zeier W.G., Janek J. (2020). Physicochemical concepts of the lithium metal anode in solid-state batteries. Chem. Rev..

[bib19] Kubota K., Nohira T., Goto T., Hagiwara R. (2008). Novel inorganic ionic liquids possessing low melting temperatures and wide electrochemical windows: binary mixtures of alkali bis (fluorosulfonyl) amides. Electrochem. Commun..

[bib20] Liu J., Gao X., Hartley G.O., Rees G.J., Gong C., Richter F.H., Janek J., Xia Y., Robertson A.W., Johnson L.R. (2020). The interface between Li_6. 5_La_3_Zr_1. 5_Ta_0. 5_O_12_ and liquid electrolyte. Joule.

[bib21] Liu L., Qi X., Ma Q., Rong X., Hu Y.S., Zhou Z., Li H., Huang X., Chen L. (2016). Toothpaste-like electrode: a novel approach to optimize the interface for solid-state sodium-ion batteries with ultralong cycle life. ACS Appl. Mater. Inter..

[bib22] Ma T., Xu G.-L., Li Y., Wang L., He X., Zheng J., Liu J., Engelhard M.H., Zapol P., Curtiss L.A. (2017). Revisiting the corrosion of the aluminum current collector in lithium-ion batteries. J. Phys. Chem. Lett..

[bib23] Mandai T., Yoshida K., Ueno K., Dokko K., Watanabe M. (2014). Criteria for solvate ionic liquids. Phys. Chem. Chem. Phys..

[bib24] Monroe C., Newman J. (2005). The impact of elastic deformation on deposition kinetics at lithium/polymer interfaces. J. Electrochem. Soc..

[bib25] Murugan R., Thangadurai V., Weppner W. (2007). Fast lithium ion conduction in garnet-type Li_7_La_3_Zr_2_O_12_. Angew. Chem. Int. Ed..

[bib26] Ni J.E., Case E.D., Sakamoto J.S., Rangasamy E., Wolfenstine J.B. (2012). Room temperature elastic moduli and Vickers hardness of hot-pressed LLZO cubic garnet. J. Mater. Sci..

[bib27] Ohta S., Seki J., Yagi Y., Kihira Y., Tani T., Asaoka T. (2014). Co-sinterable lithium garnet-type oxide electrolyte with cathode for all-solid-state lithium ion battery. J. Power Sources.

[bib28] Sharafi A., Kazyak E., Davis A.L., Yu S., Thompson T., Siegel D.J., Dasgupta N.P., Sakamoto J. (2017). Surface chemistry mechanism of ultra-low interfacial resistance in the solid-state electrolyte Li_7_La_3_Zr_2_O_12_. Chem. Mater..

[bib29] Sugata S., Saito N., Watanabe A., Watanabe K., Kim J.D., Kitagawa K., Suzuki Y., Honma I. (2018). Quasi-solid-state lithium batteries using bulk-size transparent Li_7_La_3_Zr_2_O_12_ electrolytes. Solid State Ion..

[bib30] Tatsumisago M., Nagao M., Hayashi A. (2013). Recent development of sulfide solid electrolytes and interfacial modification for all-solid-state rechargeable lithium batteries. J. Asian Ceram. Soc..

[bib31] Taylor N.J., Stangeland-Molo S., Haslam C.G., Sharafi A., Thompson T., Wang M., Garcia-Mendez R., Sakamoto J. (2018). Demonstration of high current densities and extended cycling in the garnet Li_7_La_3_Zr_2_O_12_ solid electrolyte. J. Power Sources.

[bib32] Tsai C., Roddatis V., Chandran V., Ma Q., Uhlenbruck S., Bram M., Heitjans P., Guillon O. (2016). Li_7_La_3_Zr_2_O_12_ interface modification for Li dendrite prevention. ACS Appl. Mater. Inter..

[bib33] Tsuzuki S., Shinoda W., Matsugami M., Umebayashi Y., Ueno K., Mandai T., Seki S., Dokko K., Watanabe M. (2015). Structures of [Li(glyme)]^+^ complexes and their interactions with anions in equimolar mixtures of glymes and Li [TFSA]: analysis by molecular dynamics simulations. Phys. Chem. Chem. Phys..

[bib34] Van Noorden R. (2014). The rechargeable revolution: a better battery. Nat. News.

[bib35] Wakasugi J., Munakata H., Kanamura K. (2017). Effect of gold layer on interface resistance between lithium metal anode and Li_6 25_Al_0 25_La_3_Zr_2_O_12_ solid electrolyte. J. Electrochem. Soc..

[bib36] Wang C., Gong Y., Liu B., Fu K., Yao Y., Hitz E., Li Y., Dai J., Xu S., Luo W. (2017). Conformal, nanoscale ZnO surface modification of garnet-based solid-state electrolyte for lithium metal anodes. Nano Lett..

[bib37] Wang D., Zhu C., Fu Y., Sun X., Yang Y. (2020). Interfaces in garnet-based all-solid-state lithium batteries. Adv. Energy Mater..

[bib38] Wang M.J., Choudhury R., Sakamoto J. (2019). Characterizing the Li-Solid-Electrolyte interface dynamics as a function of stack pressure and current density. Joule.

[bib39] Wang Y., Zhang Q., Xue Z.C., Yang L., Wang J., Meng F., Li Q., Pan H., Zhang J.N., Jiang Z. (2020). An in situ formed surface coating layer enabling LiCoO_2_ with stable 4.6 V high-voltage cycle performances. Adv. Energy Mater..

[bib40] Watanabe M., Thomas M.L., Zhang S., Ueno K., Yasuda T., Dokko K. (2017). Application of ionic liquids to energy storage and conversion materials and devices. Chem. Rev..

[bib41] Whittingham M.S. (1976). Electrical energy storage and intercalation chemistry. Science.

[bib42] Yamada H., Ito T., Kammampata S.P., Thangadurai V. (2020). Toward understanding the reactivity of garnet-type solid electrolytes with H_2_O/CO_2_ in a glovebox using X-ray photoelectron spectroscopy and electrochemical methods. ACS Appl. Mater. Inter..

[bib43] Yamada Y. (2020). Concentrated battery electrolytes: developing new functions by manipulating the coordination states. Bull. Chem. Soc. Jpn..

[bib44] Yoshino A. (2012). The birth of the lithium-ion battery. Angew. Chem. Int. Ed..

[bib45] Yu S., Schmidt R.D., Garcia-Mendez R., Herbert E., Dudney N.J., Wolfenstine J.B., Sakamoto J., Siegel D.J. (2016). Elastic properties of the solid electrolyte Li_7_La_3_Zr_2_O_12_ (LLZO). Chem. Mater..

[bib46] Zhao C.Z., Zhao B.C., Yan C., Zhang X.Q., Huang J.Q., Mo Y., Xu X., Li H., Zhang Q. (2020). Liquid phase therapy to solid electrolyte–electrode interface in solid-state Li metal batteries: a review. Energy Storage Mater..

[bib47] Zhao N., Khokhar W., Bi Z., Shi C., Guo X., Fan L.Z., Nan C.W. (2019). Solid garnet batteries. Joule.

[bib48] Zheng H., Wu S., Tian R., Xu Z., Zhu H., Duan H., Liu H. (2019). Intrinsic lithiophilicity of Li–garnet electrolytes enabling high-rate lithium cycling. Adv. Funct. Mater..

