# Supplementary material for: Ionic liquid-containing cathodes empowering ceramic solid electrolytes
Source: iScience. 2022 Feb 11;25(3):103896. doi: 10.1016/j.isci.2022.103896 (PMC8873615; doi:10.1016/j.isci.2022.103896)
Supplement: Document S1. Figures S1–S7 and Tables S1–S4 [file mmc1.pdf]

iScience, Volume *25*

Supplemental information

Ionic liquid-containing cathodes empowering ceramic solid electrolytes

Eric Jianfeng Cheng, Mao Shoji, Takeshi Abe, and Kiyoshi Kanamura

SUPPLEMENTAL INFORMATION

Table S1 Ionic conductivity of the Li(G4)FSI solvate IL (designated as GF), Related to Figure 2.

Temperature (°C)	Ionic conductivity (10^{-3} S cm^{-1})
30	2.1
40	3.1
50	4.0
60	6.2
70	8.0
80	10.2

Table S2 Ionic conductivity of the 1 mol dm^{-3} LiTFSI/EMI-TFSI (designated as ET), Related to Figure 2.

Temperature (°C)	Ionic conductivity (10^{-3} S cm^{-1})
30	5.0
40	6.9
50	9.5
60	12.5
70	15.9
80	19.7

Table S3 Ionic conductivity of the Al-doped LLZO pellet, Related to Figure 2.

Temperature (°C)	Ionic conductivity (10^{-4} S cm^{-1})
30	3.6
40	4.8
50	7.7
60	10.5
70	13.8
80	18.3

Table S4 IL content in the quasi-solid-state LCO cathode, Related to Figures 4 and 6.

Equimolar Li(G4)FSI solvate IL (designated as GF) series			
x	Wt%	Vol%	Cell
0	0	0	All-solid-state
5	4.8	14.6	GF 5
10	9.1	25.5	GF 10
50	33.3	63.1	GF 50
1 mol dm^{-3} LiTFSI/EMI-TFSI conventional IL (designated as ET) series			
4.3	4.1	11.1	ET 4.3
8.5	7.8	19.8	ET 8.5
12.8	11.3	27.1	ET 12.8
17	14.5	33.1	ET 17
21.3	17.6	38.2	ET 21.3

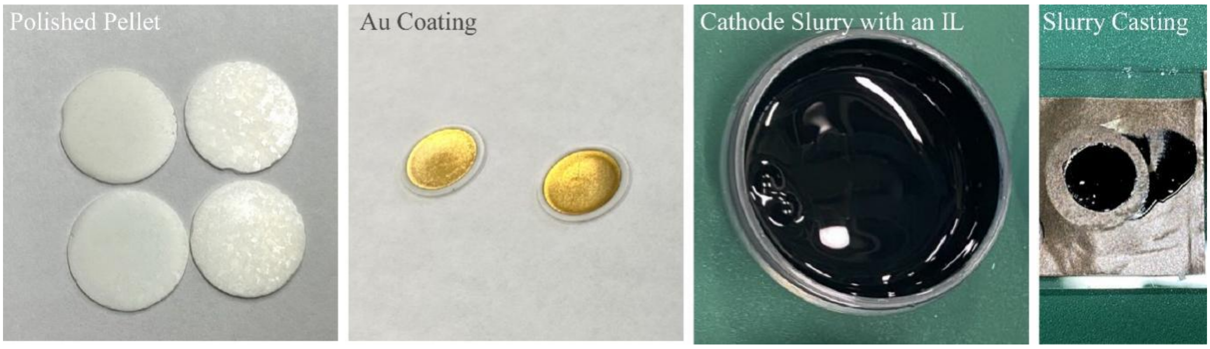

Figure S1. The procedure of casting the quasi-solid-state LCO cathode slurry onto the surface of the Al-LLZO pellet, Related to Figure 3.

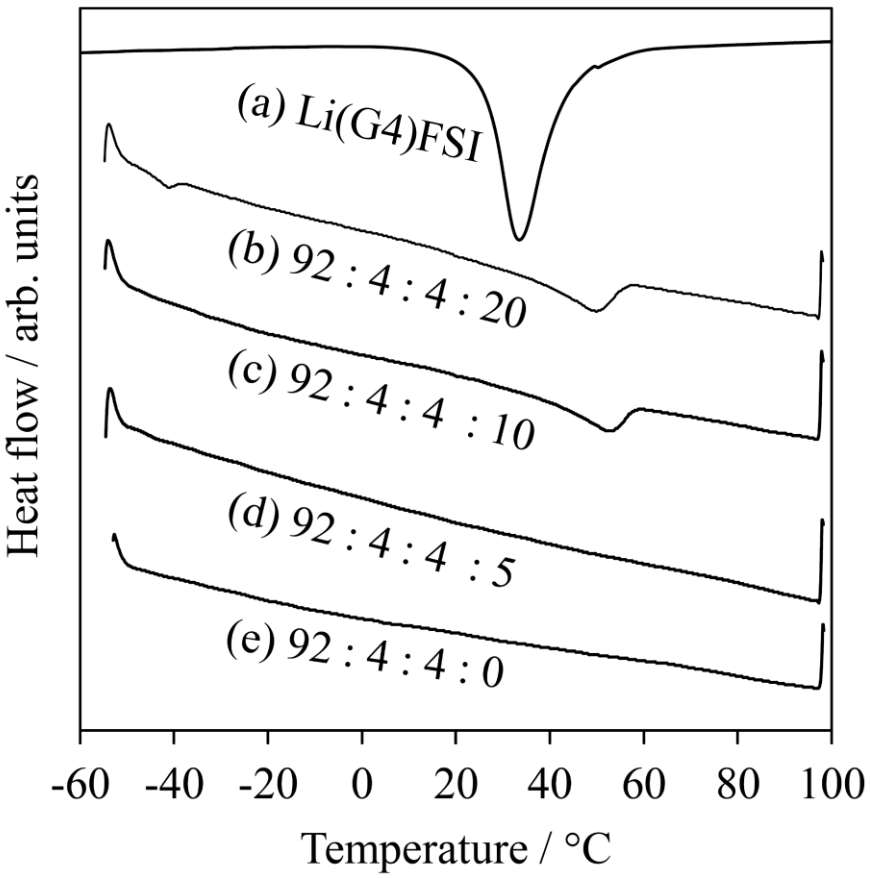

Figure S2. DSC profiles of the quasi-solid-state LCO cathode with different GF contents. The weight ratio of LCO: AB: PVDF: GF in the quasi-solid-state LCO cathode was 92: 4: 4: *x* (w/w). (a) Pure Li(G4)FSI (GF), (b) *x*=20, (c) *x*=10, (d) *x=5*, and (e) *x*=0, Related to Figure 3.

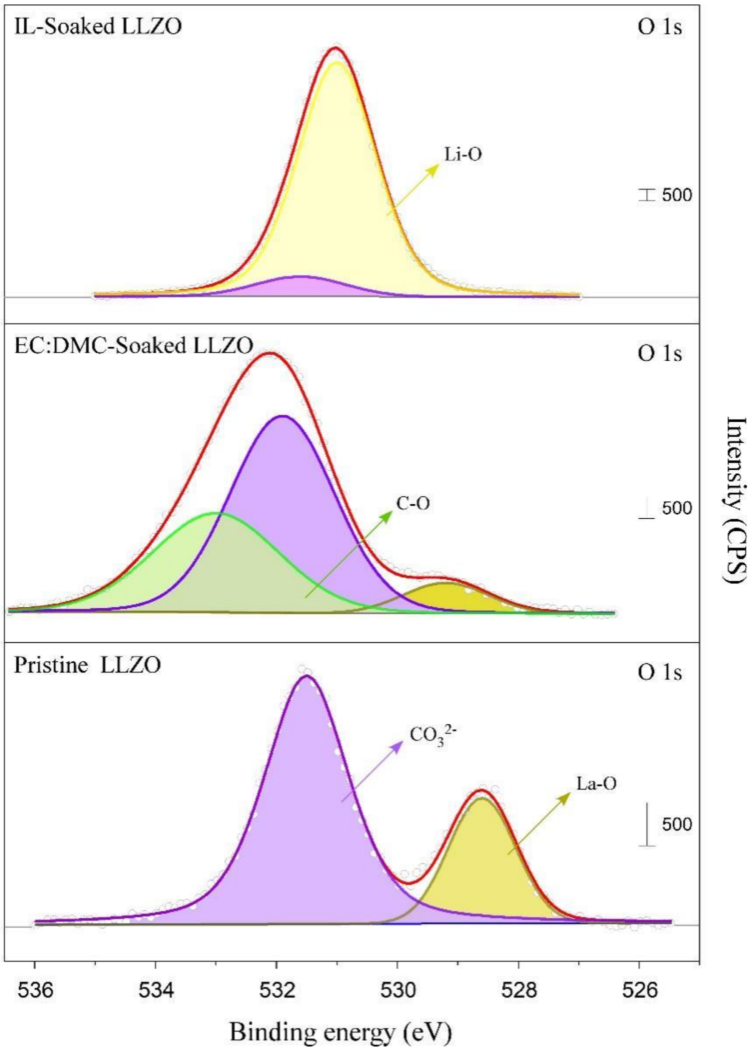

Figure S3. Analysis of the surface chemistry (O 1s) of the Al-LLZO pellet in different conditions. As-polished (pristine), soaked in 1 mol dm^{-3} $LiPF_6$/EC-DMC (v:v=1:1) (LP30) for 200 h and soaked in the conventional ET IL for 200 h, Related to Figure 8.

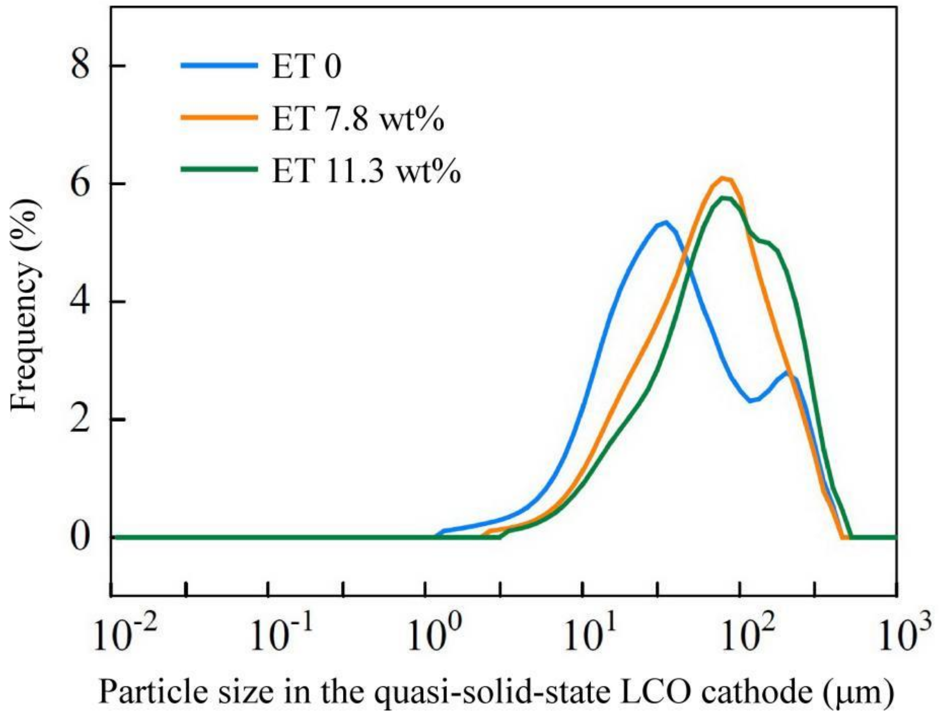

Figure S4. DLS analysis of the particle size in the quasi-solid-state LCO cathode as a function of the IL content. The IL here is the conventional 1 mol dm^{-3} LiTFSI/EMI-TFSI and its content in the quasi-solid-state LCO cathode was 0, 7.8 wt%, and 11.3 wt%, respectively. The particle size in the quasi-solid-state LCO cathode increases with increasing IL content, Related to Figure 11.

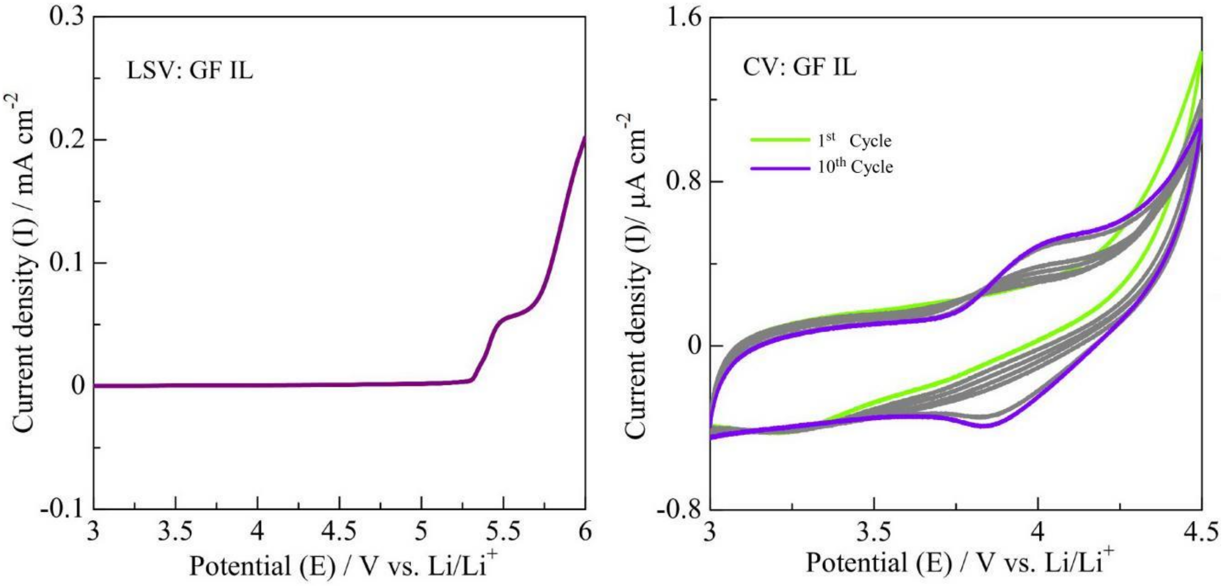

Figure S5. Electrochemical stability analysis of the solvate GF IL at 25 °C. (a) Linear sweep voltammogram, (b) Cyclic voltammogram. The scan rates for LSV and CV were 1 mV S^{-1}. An Al foil was used as the working electrode (WE), and a Li foil was used as the counter electrode (CE) as well as the reference electrode (RE). A highly porous polyimide film (PI) was used as the separator. Broad redox peaks are observed at about 3.8 V and 4.0 V *vs*. Li/Li^+ in the CV profiles and the processes look reversible. They are likely related to impurity phases on the surface of the Al working electrode or a slow corrosion process of the Al working electrode in the IL, Related to Figure 11.

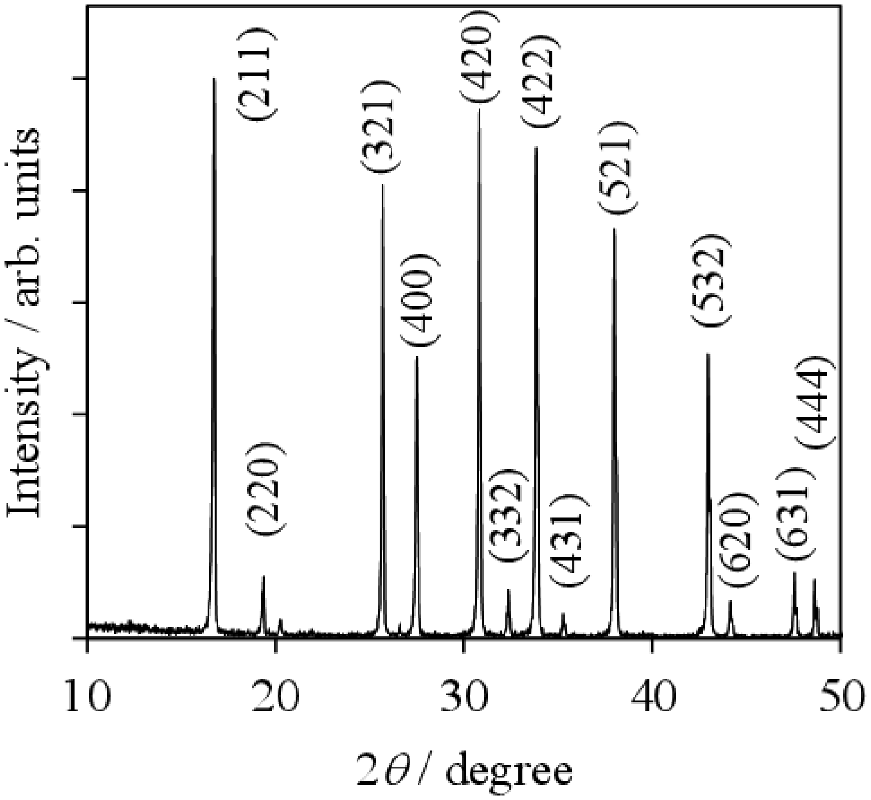

Figure S6. XRD profile of the 1000 °C sintered Al-LLZO pellet. It indicates that the high temperature-sintered Al-LLZO pellet has a cubic structure, Related to Figure 2.

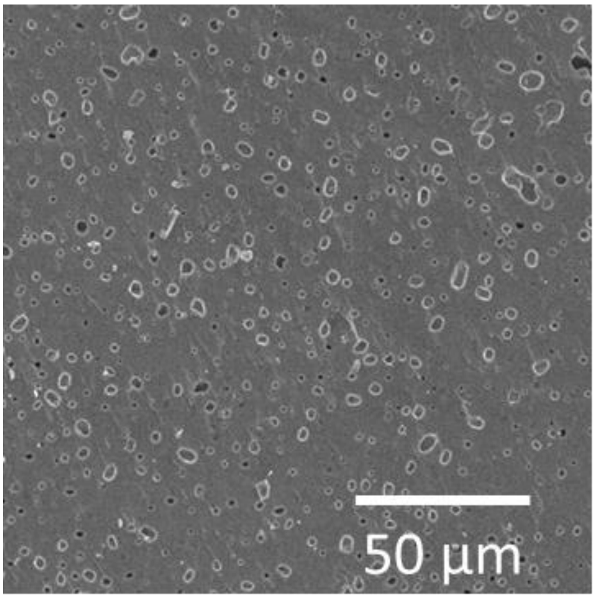

Figure S7. A cross-sectional SEM micrograph of the 1000 °C sintered Al-LLZO pellet. Micropores are observed and the relative density of the Al-LLZO pellet is about 95%, Related to Figure 2.
